# CD39 and immune regulation in a chronic helminth infection: The puzzling case of *Mansonella ozzardi*

**DOI:** 10.1371/journal.pntd.0006327

**Published:** 2018-03-05

**Authors:** Nathália F. Lima, Raquel M. Gonçalves-Lopes, Yvonne C. M. Kruize, Maria Yazdanbakhsh, Marcelo U. Ferreira

**Affiliations:** 1 Department of Parasitology, Institute of Biomedical Sciences, University of São Paulo, São Paulo, Brazil; 2 Department of Parasitology, Leiden University Medical Center, Leiden, The Netherlands; CSIR-Indian Institute of Chemical Biology, INDIA

## Abstract

**Background:**

Chronic helminth infections typically induce an immunoregulatory environment, with markedly reduced immune responses to both parasite-specific and unrelated bystander antigens. Here we tested whether these changes are also observed in human infections with *Mansonella ozzardi*, a neglected filarial nematode widely distributed across Latin America.

**Methods:**

CD4^+^ T cell populations from microfilaremic (Fil+) and uninfected (Fil-) inhabitants in *M*. *ozzardi*-endemic riverine communities in Brazil were characterized by flow cytometry analysis. Plasma concentrations of a wide range of cytokines and chemokines were measured. We examined whether *M*. *ozzardi* infection is associated with suppressed *in vitro* lymphoproliferative and inflammatory cytokine responses upon stimulation with filarial antigen, unrelated antigens or mitogens.

**Principal findings/Conclusions:**

Fil+ subjects had lower plasma levels of selected inflammatory cytokines, such as TNF-α, IL-8, and IL-6, than their Fil- counterparts. However, we found no evidence for attenuated T-cell responses to filarial antigens or co-endemic pathogens, such as malaria parasites and *Toxoplasma gondii*. CD4^+^ T cells expressing CD39, an ectonucleosidase involved in the generation of the anti-inflammatory molecule adenosine, were increased in frequency in Fil+ subjects, compared to uninfected controls. Significantly, such an expansion was directly proportional to microfilarial loads. Surprisingly, CD39 blocking with a neutralizing antibody suppressed antigen-driven lymphoproliferation *in vitro*, while decreasing inflammatory cytokine responses, in Fil+ and Fil- individuals. These findings suggest that circulating CD4^+^ CD39^+^ T cells comprise subsets with both regulatory and stimulatory roles that contribute to the immune homeostasis in chronic *M*. *ozzardi* infection.

## Author summary

Helminth infections downregulate immunity and reduce host’s inflammatory responses, but the filarial nematode *Mansonella ozzardi*, which is widely distributed across Latin America, appears to represent an exception to this rule. We found similar lymphoproliferative responses to filarial and unrelated antigens and comparable regulatory cytokine responses in subjects harboring *M*. *ozzardi* microfilariae, compared to local uninfected controls. The proportion of CD4^+^ T cell subtypes expressing CD39 was significantly increased in infected subjects and correlated positively with their microfilarial density. However, antibody blocking of CD39, an ectonucleosidase involved in the synthesis of the immunosuppresive molecule adenosine, paradoxically reduced, rather than promoted, antigen-driven lymphoproliferation *in vitro*. We suggest that CD39^+^ CD4^+^ T cells circulating in microfilaremics comprise both regulatory and stimulatory cell subsets that are concomitantly expanded. The balance between these cell subsets with opposing regulatory functions may be crucial to maintain immune homeostasis during chronic *M*. *ozzardi* infections.

## Introduction

Helminth parasites induce a wide range of mechanisms that downregulate immunity, limiting host’s inflammation-mediated tissue damage and favoring parasites’ persistence in typically asymptomatic carriers [1,2]. Nearly 40 years ago, T cell proliferation responses to parasite-specific antigens, but not to mitogens, were first shown to be severely dampened in chronic human infections with tissue-dwelling helminths, such as in schistosomiasis and lymphatic filariasis [3]. Likewise, reduced antigen-driven T-cell responses have also been documented in infections with lumen-dwelling intestinal nematodes [4]. The immunomodulatory environment in these long-lasting infections is characterized by cytokine responses skewed towards interleukin (IL)-10 and transforming growth factor (TGF)-β and the expansion of several regulatory T (Treg) and B cell subtypes, including the classical CD4^+^CD25^hi^CD127^-^FoxP3^+^ Treg cells. Immune responses to unrelated bystander antigens, such as co-occurring pathogens, allergens, and vaccines, are also often suppressed—a phenomenon termed spillover suppression [5]. Because major infectious diseases such as HIV infection, tuberculosis, and malaria are endemic to areas where several helminth infections are highly prevalent, spillover suppression has major public health implications [5]. However, it remains unclear to what extent similar regulatory networks operate in all chronic helminth infections of humans.

Three filarial nematodes of the genus *Mansonella* infect humans: *M*. *streptocerca*, which is endemic to Africa; *M*. *perstans*, which is commonly found in Africa but also occurs in South America [6]; and *M*. *ozzardi*, which is found exclusively in Latin America and the Caribbean islands [7]. *M*. *ozzardi* has a patchy geographic distribution from southern Mexico to northwestern Argentina, with overall prevalence ranging from <1% to 46% in affected communities [8,9]. Adult worms have been recovered from subcutaneous tissues of experimentally infected patas monkeys [10], but their habitat in human hosts remains uncertain. Microfilariae circulate in the peripheral blood all day long, with very little, if any, periodicity. Most human infections cause little or no disease and may remain undiagnosed and untreated for several years [8].

Infections with the filarial nematodes *Wuchereria bancrofti*, *Brugia malayi*, and *M*. *perstans* typically reduce immunity to malaria parasites and other pathogens, with an IL-10-dependent decrease in IL-12p70, interferon (IFN)-γ and IFN-γ-induced protein 10 (IP-10) responses upon T-cell stimulation [11–14]. Nevertheless, the immunomodulatory potential of *M*. *ozzardi* infections and their spillover effects remain largely unexplored—even in the Amazon Basin, where this filarial nematode and malaria parasites are endemic and co-infections with them are very likely.

Here we hypothesized that immune responses might be also downregulated in subjects chronically harboring *M*. *ozzardi* microfilariae. We first characterized peripheral blood mononuclear cell (PBMC) populations from people living in *M*. *ozzardi*-endemic communities of Brazil and found an expansion of CD4^+^ T cell subtypes expressing CD39 in infected subjects, compared to local uninfected controls. CD39 is an ectonucleosidase that catalyzes the phosphohydrolysis of extracellular ATP and ADP to AMP, which is in turn used by CD73 to synthesize the immunosuppresive molecule adenosine [15]. We next examined whether *M*. *ozzardi* infection is associated with suppressed T-cell responses driven by filarial and unrelated antigens and whether this is mediated through the induction of high levels of regulatory cytokines. Finally, we explored the effects of *in vitro* neutralization of CD39 expression on antigen-driven lymphoproliferative responses and cytokine production.

## Methods

### Ethics statement

Study protocols were approved by the Institutional Review Board of the Institute of Biomedical Sciences, University of São Paulo, Brazil (1133/CEP, 2013). Written informed consent was obtained from all patients or their parents or guardians, if participants were minors aged <18 years.

### Study area

The field study was carried out in six villages—Monte Verde, Valparaíso, Boa Vista, Retiro, Nova Vida, and São Pedro—situated along the banks of Purus River, in northwestern Brazil (S1 Fig). These villages are located in the municipality of Boca do Acre (8°45’19"N, 67°23’50"W), southern Amazonas state, with a combined population of 1,300 inhabitants. They had previously been shown to be endemic for *M*. *ozzardi*, with an average prevalence of infection estimated at 27.3% [16]. Although *M*. *ozzardi* vectors have not been characterized in this area, simuliid black flies of the species *Simulium amazonicum*, *S*. *argentiscutum*, *S*. *oyapockense s*.*l*., and *S*. *roraimense* are believed to transmit this helminth across the Amazon Basin of Brazil [17]. Low-level malaria transmission is recorded year-round in these communities, with *Plasmodium vivax* accounting for over 95% of the infections in 2013. Other tissue-dwelling helminths such as *Schistosoma mansoni* and *W*. *bancrofti* are not endemic to this region.

During a pilot study in March 2013, we carried out house-to-house visits in the target communities and randomly screened 287 inhabitants for *M*. *ozzardi* microfilariae. By combining thick-smear microscopy and polymerase chain reaction (PCR) on finger-prick blood samples, we found 41 (14.3%) subjects harboring *M*. *ozzardi* microfilariae, with prevalences of infection ranging between 6.1% and 30.4% across communities. As described in similar endemic settings [18], prevalence of infection increased with age, from 4.4% in children below 10 years to 57.1% in adults over 50 years.

### Study population

During the next visit to the target communities, in September 2013, we invited subjects previously found to harbor microfilariae, as well as a subsample of individuals found to be uninfected or not screened during the pilot survey, to contribute 40 mL of venous blood for microfilariae detection and PBMC and plasma separation. Because most infected subjects were adults, we prioritized adults as uninfected controls in order to have a similar age composition in the comparison groups. Blood was drawn between 9:00 am and 3:00 pm. Infections diagnosed during the pilot study and those newly diagnosed during this second survey were treated with a single dose of 0.2mg/kg of ivermectin [19] after blood sampling. No post-treatment samples were obtained.

Study subjects were given plastic containers containing 10% formalin and asked to provide a stool sample. Stool specimens were examined for eggs, cysts and larvae of intestinal parasites with a standard sedimentation method [20], which was preferred over the standard Kato-Katz method because we used formalin-diluted stool samples. No study participant had intestinal parasites detected by stool examination; furthermore, none of them had malaria diagnosed by using a quantitative real-time PCR targeting the 18S rRNA gene [21]. Plasma samples from all study participants were screened for IgG antibodies to *Toxoplasma gondii* by using the Serion ELISA classic IgG kit (Institut Viron/Serion, Würzburg, Germany); all of them were seropositive.

### *Mansonella ozzardi* microfilaremia and filarial IgG_4_ antibodies

Laboratory diagnosis of *M*. *ozzardi* was based on microscopic examination of thick smears and quantitative PCR on all samples. Subjects positive by either method were defined as microfilaremic (Fil+) and those negative by both methods were defined as Fil-. Thick blood smears were stained with Giemsa; at least 100 fields were examined for microfilariae, under 1000 × magnification, before a slide was declared negative. We standardized an in-house, SYBR green-based real-time PCR approach to quantify the number of copies of amplicons, which was used as a proxy of microfilarial density. DNA templates for PCR amplification were isolated from 200 μL of whole venous blood on a QIAcube automated platform (Qiagen, Hilden, Germany), using QIAamp DNA blood kits (Qiagen), and eluted in 200 μL for water. Each 20-μL reaction mixture contained 2 μL of sample DNA (corresponding to 2 μL of whole blood), 10 μL of 2× Maxima SYBR Green qPCR master mixture (Fermentas, Burlington, Canada) and 0.5 μM of each primer (forward, 5’-CTT ATC ATC AGG TGA TAT TAA T-3’; reverse, 5’-TTA GTT TCT TTT CCT CCG CT-3’). These primers allow the amplification of a *M*. *ozzardi*-specific 295-base pair (bp) fragment of the internal transcribed spacer (ITS)-2 of the ribosomal DNA (rDNA) gene [22]. Standard curves were prepared with serial tenfold dilutions of the target sequence, cloned into pGEM-T Easy vectors (Promega, Madison, WI, USA), to allow for calculating the number of ITS-2 amplicons/μL of blood. We used a Mastercycler gradient real-time PCR cycler (Eppendorf, Hamburg, Germany) for PCR amplification with a template denaturation step at 95°C for 10 min, followed by 40 cycles of 15 sec at 95°C and 1 minute at 55°C, with fluorescence acquisition at the end of each extension step. Amplification was immediately followed by a melting program consisting of 15 sec at 95°C, 15 sec at 55°C, and a stepwise temperature increase of 0.2°C/sec until 95°C, with fluorescence acquisition at each temperature transition. No-template controls (containing all reagents for amplification except for the DNA template) were run for every qPCR microplate.

Filaria-specific IgG_4_ antibodies were determined by ELISA as described [23], using a *B*. *malayi* adult worm extract (BmA) as a solid-phase capture antigen.

### Plasma cytokines

We compared plasma levels of the following cytokines in Fil+ and Fil- subjects: epidermal growth factor (EGF), eotaxin, fibroblast growth factor (FGF)-basic, granulocyte-colony stimulating factor (G-CSF), granulocyte-macrophage colony-stimulating factor (GM-CSF), hepatocyte growth factor (HGF), IFN-α, IFN-γ, IL-1ra, IL-1β, IL-2, IL-2r, IL-4, IL-5, IL-6, IL-7, IL-8, IL-10, IL-12 (p40/p70), IL-13, IL-15, IL-17, IP-10, monocyte chemoattractant protein (MCP)-1, monokine induced by IFN-γ (MIG, also known as CXCL9), macrophage inflammatory protein (MIP)-1α, MIP-1β, CCL5 (also known as RANTES, Regulated on Activation, Normal T Cell Expressed and Secreted), TNF-α, and vascular endothelial growth factor (VEGF). Samples were tested using the Human Cytokine Magnetic 30-Plex Panel (Invitrogen), and data were acquired with a Bio-Plex 200 System (Bio-Rad) using Luminex 100 xMAP technology (Luminex, Austin, USA).

### Peripheral blood mononuclear cell separation and phenotyping

PBMC were separated by gradient centrifugation with Ficoll-Paque Plus (GE Healthcare, Little Chalfont, United Kingdom), up to 6 hours after blood collection, cryopreserved in liquid nitrogen, and thawed as described [24]. PBMC were evaluated for viability and only samples with >90% viability were used in subsequent analyses.

CD4^+^ T cell subtypes circulating in the peripheral blood of Fil+ and Fil-subjects were defined according to the (co-)expression of regulatory and activation markers. Regulatory molecules included: (a) the transcription factor FoxP3, (b) the TNF receptor family costimulatory receptor OX-40 (CD134), (c) the glucocorticoid-inducible TNF receptor family-related protein (GITR/CD357), (d) the TNF receptor II (TNFRII/CD120b), (e) the ectonucleosidase CD39 (also known as NTPDase1), (f) the programmed cell death protein 1 (PD-1/ CD279), (g) the lymphocyte-activation gene 3 product (LAG-3/CD223), (h) the membrane-bound C-terminal pro-region of TGF-β known as latency-associated peptide (LAP-TGF-β), and (i) the primary inhibitory receptor CTLA-4 (CD152), which competes with CD28 for CD80 and CD86 binding on antigen-presenting cells. The activation markers were: (a) HLA-DR, which identifies a Treg cell subset involved in contact-dependent immune suppression, and (b) CD69, an early activation marker with inhibitory properties. Treg cells were phenotypically defined as CD4^+^CD25^hi^ cells that express FoxP3 but not the α chain of the IL-7 receptor (CD127).

After thawing, 10^6^ viable cells/mL were transferred to V-bottomed 96-well microplates with 200 μL of staining buffer (PBS with 2% fetal bovine serum and 2 mm EDTA) per well and centrifuged at 600 × g for 5 min. For surface staining, the cell pellet was incubated with directly conjugated antibodies at room temperature, in the dark, for 30 min and washed twice with 200 μL of staining buffer. To analyze the intracellular expression of FoxP3 and Ki67, a nuclear protein that regulates cell division, cells were fixed and permeabilized using the Transcription Factor Fixation/Permeabilization kit of eBiosciences (San Diego, CA, USA) and then incubated with specific, labeled antibodies. In this context, we used intracellular Ki67 expression as an indicator of T-cell proliferation [25]. Monoclonal antibody panels are listed in S1, S2 and S3 Tables. Samples were acquired on an LSR Fortessa flow cytometer (BD Biosciences, San Jose, CA, USA) using the FACSDiva software (BD Biosciences). Anti-mouse IgG-coated beads (BD Biosciences) stained with each fluorochrome separately were used for software-based compensation. We used the Live/Dead Fixable Blue Dead Cell Stain for UV excitation (Invitrogen, Carlsbad, CA, USA) for dead cell exclusion and collected ≥ 300,000 events (panels 1 and 2, S1 and S2 Tables) or ≥ 600,000 events (panel 3, S3 Table) in each live gate. Only samples with >80% viability were analyzed. Fluorescence minus one (FMO) controls were used to control for spectral overlap. Boolean analysis was applied to evaluate co-expression of different molecules by the same cells. Data analysis was carried out using FlowJo software version 8.8.6 (Tree Star, Ashland, OR, USA).

### Antigens and PBMC proliferation assays

We used BmA, prepared as described [26], to elicit filarial-specific lymphocyte proliferation *in vivo*. As bystander antigens, we used: (a) commercially available *Staphylococcus aureus* enterotoxin B (SEB; Sigma-Aldrich), (b) a soluble tachizoite extract prepared with the RH strain of *T*. *gondii* (TgT) [27], and (c) red blood cells (RBC) infected with *P*. *vivax* schizonts (PvS). To prepare PvS, patient-derived infected blood was filtered through Fresenius Kabi BioR01 Plus filters (Bad Homburg, Germany) to remove WBC and cultured for 44–46 h in McCoy’s 5A medium (Invitrogen) supplemented with 20% human AB serum until intracellular parasites reached the mature schizont stage (≥ 4 nuclei) [28]. RBC infected with mature schizonts were enriched by using MACS separation columns (Miltenyi, Auburn, CA, USA) as described [29].

To examine whether *M*. *ozzardi* is associated with T-cell hyporesponsiveness, we stimulated PBMC from Fil+ and Fil- subjects with the antigens described above. To this end, 10^6^ PBMC/well were labelled with CellTrace Violet (Invitrogen) and incubated in 96-well microplates with either BmA (13 μg/mL), SEB (10 μg/mL), TgT (12 μg/mL), or 10^5^ *P*. *vivax*-infected RBC/well, to a final volume of 200 μL/well. BmA and SEB were used with 10 μg/mL of anti-CD28/CD49d co-stimulus (BD FastImmune, San Jose, CA, USA). Cells were incubated for 72 h at 37°C in RPMI-1640 medium supplemented with 10% inactivated fetal bovine serum, 10 mM HEPES, 2 mM L-glutamine, 1 mM sodium pyruvate, 55 μM 2-mercaptoethanol, and a 1% (vol/vol) solution containing 100 U/mL of penicillin, 10 μg/mL of streptomycin, and 25 μL/mL of amphotericin B in a humidified chamber with 5% CO_2_. Medium alone and 10 μg/mL of phytohemagglutinin (PHA-P, Sigma-Aldrich, St. Louis, MO, USA) were used as negative and positive controls, respectively. After acquisition of ≥ 300,000 events in each live gate on an LRS Fortessa or FACSCanto II flow cytometer (BD Biosciences), the proportion of PBMC that had divided at least once during the culture period was calculated using the FlowJo software. Here, we subtracted the proportion of divided PBMC in medium-only wells (“unstimulated”) from that of antigen-containing wells to obtain net proportions of divided cells. Only samples with >80% viability were analyzed.

To test the effect of *in vitro* CD39 blocking on lymphoproliferation, we compared the proportion of dividing PBMC after stimulation with SEB (10 μg/mL) and anti-CD28/CD49d (10 μg/mL), as described above, in the absence (medium alone) or the presence of 2 μg/mL of anti-CD39 antibody (Biolegend, San Diego, CA, USA) [30]. At this concentration, this antibody was previously shown, by flow cytometry, to abolish surface CD39 recognition by the FITC-labeled anti-CD39 monoclonal antibody used in PBMC phenotyping. As a positive control, we used 2 μg/mL of a neutralizing anti-IL-10 antibody (Biolegend). Separate lymphoproliferation assays were carried out in parallel with the addition of 2 mM ATP or adenosine (both from Sigma-Aldrich) to cells that had been pre-incubated with the blocking anti-CD39 antibody.

### Cytokine production after PBMC stimulation *in vitro*

We used standard intracellular cytokine staining to evaluate the production and accumulation, after antigen stimulation, of Th1- and Th2-type cytokines within the endoplasmic reticulum of CD4^+^ cells from Fil+ and Fil- subjects. To this end, 0.5 × 10^6^ PBMC/well were cultured in U-bottomed 96-well microplates for 4 hours at 37°C in the absence (medium alone) or the presence of BmA (13 μg/mL) or SEB (10 μg/mL) combined with anti-CD28/CD49d co-stimulus (10 μg/mL). We next added 10 μg/mL of brefeldin A, to retain the cytokines within the PBMC, followed by further 19 hours at 37°C in a CO_2_ incubator. To evaluate how CD39 blocking affected cytokine production by CD4^+^ T cells, 2 μg/mL of purified anti-CD39 antibody (Biolegend) were added to selected wells shortly after antigen stimulation. Next, PBMC were transferred to V-bottomed 96-well microplates, for cell surface staining with anti-CD3 and anti-CD4 monoclonal antibodies for 30 min, followed by incubation with the permeabilization buffer supplied by eBioscience, for 1 hour at room temperature, and staining with monoclonal antibodies. The monoclonal antibody panel used for intracellular staining of IL-2, TNF-α, IFN-γ, IL-4, IL-5, and IL-13 is listed in S4 Table. We used a FACSCanto II flow cytometer (BD Biosciences) to acquire ≥ 300,000 events in each live gate, in samples with >80% viability, and FlowJo software for data analysis.

We also evaluated cytokine levels in culture supernatants harvested after 72-h incubation of PBMC with BmA (13 μg/mL) or SEB (10 μg/mL) combined with anti-CD28/CD49d (10 μg/mL), or medium alone. Again, 2 μg/mL of purified anti-CD39 antibody (Biolegend) were added to selected wells after antigen stimulation. We used the PeliKine compact ELISA kit (Sanquin, Amsterdam, Netherlands) to measure IL-6, IL-13, IL-10, IL-4, and IFN-γ levels.

### Statistical analysis

Because most continuous variables had an overdispersed distribution, results were summarized as medians and interquartile ranges (IQR). Comparisons between samples from different subjects were done with nonparametric Mann-Whitney *U* tests (for continuous variables) or χ^2^ tests (for proportions). Paired data were compared with Wilcoxon signed rank tests for continuous variables. Nonparametric correlation coefficients (*r*_s_) were estimated using Spearman rank correlation tests. All analyses were performed using SPSS 17.0 software (SPSS, Chicago, IL, USA). Significance was defined at the 5% level. We used the Benjamini-Hochberg procedure [31] to control for the false discovery rate (*q*) when many comparisons were conducted in the same sample set and one or more of these tests resulted in a significant difference. We ranked all individual *P* values from the smallest to the largest and compared each of them to its Benjamini-Hochberg critical value, given by (*i*/*m*)*q*, where *i* is the rank and *m* is the total number of tests. We set *q* at 0.10, meaning that we accept up to 10% of the associations with significant results being false positives. The largest *P* value that has *P*<(*i*/*m*)*Q* was considered significant, as well as all of the *P* values smaller than it. Calculations of the Benjamini-Hochberg critical value were done using the Excel spreadsheet available at: http://www.biostathandbook.com/multiplecomparisons.html.

## Results

### Characteristics of study participants

Fifty microfilaremic (Fil+) subjects aged between 10 and 98 years and 34 uninfected (Fil-) controls from the same communities aged between 7 and 84 years contributed plasma and PBMC samples. The number of ITS-2 amplicons ranged between 1,300 and 2,973,350 copies/μL in microfilaremics. Fil+ and Fil- subjects did not differ significantly according to their age and sex distribution, hemoglobin levels, total IgE levels and counts of white blood cells (WBC), lymphocytes, T lymphocytes, and T CD4+ lymphocytes (Table 1). Moreover, clinical signs and symptoms were reported in comparable frequencies by Fil+ and Fil- individuals (S5 Table).

**Table 1:** Demographic, hematologic, and clinical characteristics of microfilaremic subjects (Fil+) and uninfected controls (Fil-).

Characteristic	Value for group		*P* value
	Fil-	Fil+	
No. of subjects	34	50	
Age in years (range)	37.1 (7–84)	43.8 (10–98)	0.207
Gender (% male)	52.9	56.0	0.722
Village			
Boa Vista	4	4	0.013
Monte Verde	11	5	
Nova Vida	11	11	
Retiro	3	14	
São Pedro	0	4	
Valparaíso	5	12	
Hemoglobin levels (g/100 mL)	14.5 (11.8–21.2)	14.2 (11.7–18.40)	0.665
Anemia (%)	2.9 (n = 1)	6.0 (n = 3)	0.410
No. of WBCs (10^9^/L)	7.8 (4.7–11.9)	8.2 (3.9–17)	0.658
No. of lymphocytes (10^9^/L) No. of granulocytes (10^9^/L)	2.5 (1.3–3.5) 4.6 (2.2–9.10)	2.3 (1.2–4.1) 5.0 (1.9–43)	0.193 0.266
No. of T lymphocytes (10^9^/L)	1.74 (0.94–2.64)	1.54 (0.66–3.58)	0.271
No. of CD4^+^ T lymphocytes (10^9^/L)	0.98 (0.43–1.71)	0.96 (0.43–2.13)	0.381

Data are presented as median (interquartile range) except if otherwise indicated and were compared with the Mann-Whitney test (continuous variables) or with the χ^2^ test (proportions).

IgG_4_ antibodies to BmA were measured by ELISA in available plasma samples from 82 study participants. These were significantly higher in microfilaremics than in uninfected controls (median, 3594 pg/mL vs. 1628 pg/mL, Mann-Whitney *U* test, *P* = 0.004). Twenty-six subjects, hereafter termed IgG_4_H, had BmA antibody levels above the overall median; 25 of them were microfilaremic. The IgG_4_L group comprised of 56 subjects (25 of them infected) with BmA antibody levels below the overall median. Therefore, half of the 50 Fil+ subjects but only one of the 32 Fil- subjects tested for antibodies were in the IgG_4_H group. Interestingly, the IgG_4_H and IgG_4_L groups had similar age and sex distribution, hemoglobin levels, and WBC counts, but the IgG_4_H population had significantly lower counts of lymphocytes, T lymphocytes, and T CD4+ lymphocytes (S6 Table). Specific IgG_4_ antibody concentrations in microfilaremics correlated positively with ITS-2 amplicon copy numbers (*r*_s_ = 0.315, *P* = 0.004).

### Inflammatory and regulatory cytokines in microfilaremics

Microfilaremics had significantly lower plasma concentrations of TNF-α (median, 9.7 vs. 18.4 pg/mL, *P* = 0.012), IL-4 (median, 0.0 vs. 9.2 pg/mL, *P* = 0.008), IL-8 (median, 4.7 vs. 28.5 pg/mL, *P* = 0.005), G-CSF (median, 52.4 vs. 101.9 pg/mL, *P* = 0.016), IL-6 (median, 3.8 vs. 8.0 pg/mL, *P* = 0.021), and MIP-1α (median, 16.1 vs. 28.9 pg/mL, *P* = 0.016), and significantly higher levels of the Th2-type mediator eotaxin (median, 47.7 vs. 37.0 pg/mL, *P* = 0.006), compared with uninfected controls (S7 Table). However, the slight difference between Fil+ and Fil- subjects in IL-10 concentrations (median, 17.4 vs. 13.7 pg/ml) did not reach statistical significance (*P* = 0.052). Levels of eotaxin, but not those of other cytokines or chemokines, were significantly correlated to the proportion of circulating CD4^+^ cells that were CD39^+^ (*r*_S_ = 0.356, *P* = 0.002) and to the proportion of circulating Treg cells that that were CD39^+^ (*r*_S_ = 0.274, *P* = 0.022).

### Lymphoproliferative and cytokine responses following antigen stimulation in Fil+ and Fil- subjects

**Fig 1:**
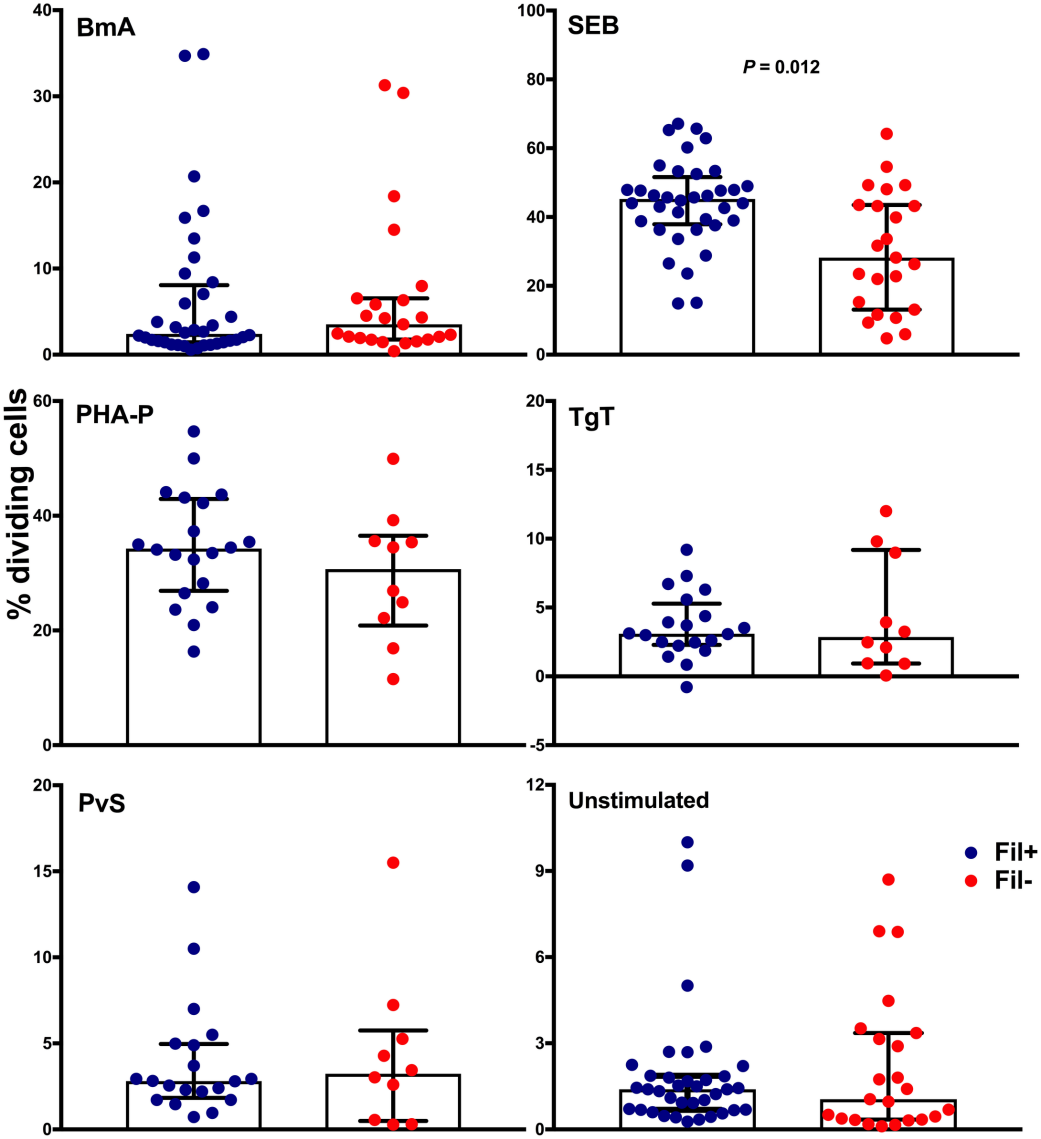
Antigen-driven lymphocyte proliferation is not suppressed in *Mansonella ozzardi* infection. PBMC from microfilaremic (Fil+) and uninfected (Fil-) subjects were labeled with CellTrace Violet and incubated with filarial (*B*. *malayi* adult worm extract [BmA]) and unrelated antigens (*Staphylococcus aureus* enterotoxin B [SEB], *Toxoplasma gondii* taquizoite extract [TgT] and red blood cells infected with *Plasmodium vivax* schizonts [PvS]), mitogen (phytohemagglutinin [PHA-P]), or medium alone (“unstimulated”). The net % of divided cells (stimulated minus unstimulated) was estimated by flow cytometry and shown in all panels, except for the lower right panel, which shows % of unstimulated divided cells. Data are presented as medians and interquartile ranges for 20 Fil+ and 10 Fil- subjects (TgT, PvS, and PHA-P) or 36 Fil+ subjects and 23 Fil- subjects (BmA, SEB, and medium alone) and were compared using the Mann-Whitney *U* test. The only significant *P* value after controlling for a false discovery rate (*q*) set at 0.10 is shown (number of comparisons *m* = 6).

We found no evidence for lymphocyte hyporesponsiveness in *M*. *ozzardi* infection. Fig 1 shows comparable proliferation patterns of PBMC from microfilaremic and uninfected subjects that were stimulated with filarial (BmA) and two bystander antigens (TgT and PvS), as well as with mitogen (PHA-P). However, SEB-driven PBMC proliferation was significantly *increased*, rather than decreased, in microfilaremics, with median proportion of dividing cells of 45.2% vs. 31.7% (*P* = 0.012) in Fil+ and Fil- subjects, respectively. Moreover, antigen-driven cytokine responses did not differ significantly between infected and uninfected subjects. We found comparable proportions of CD4^+^ T cells from Fil+ and Fil- individuals producing IFN-γ, IL-2, IL-10, TNF-α, and Th2-type cytokines (IL-4, IL-5, and IL-13) *in vitro* following stimulation with BmA or SEB (S8 Table). Finally, we found similar concentrations of secreted IL-6, IL-13, IL-10, IL-4, and IFN-γ in PBMC culture supernatants from Fil+ and Fil- subjects stimulated with BmA and SEB (S9 Table).

### Higher frequency of CD39-expressing CD4^+^ T cells and Treg cells in Fil+ subjects

We compared the expression of regulatory and activation markers in circulating CD4^+^ T cells from Fil+ and Fil- subjects, using the gating strategy shown in S2 and S3 Figs. Fil+ and Fil- subjects had similar frequencies of CD4^+^ T cells expressing most regulatory and activation markers (Table 2). However, microfilaremics had a significantly higher frequency of CD4^+^ T cells expressing CD39 than uninfected controls (median, 7.2% vs. 5.5% of all circulating CD4^+^ T cells). A similar difference in the frequency of CD4^+^ T cells expressing CD39 was observed when comparing IgG_4_H and IgG_4_L subjects (S10 Table).

**Table 2:** Frequency of CD4^+^ T cells expressing regulation and activation markers in microfilaremic subjects (Fil+) and uninfected controls (Fil-).

Marker	Value for group (% of CD4^+^ T cells)		*P* value
	Fil-	Fil+	
No. of subjects	33	48	
CD39	5.52 (3.89–7.34)	7.23 (5.58–9.23)	0.005*
CTLA-4	0.56 (0.22–3.32)	0.52 (0.18–5.55)	0.338
Intracellular CTLA-4	21.60 (10.90–31.40)	20.00 (10.30–41.20)	0.839
HLA-DR	4.30 (2.34–6.91)	4.89 (2.50–8.18)	0.131
PD-1	9.12 (5.55–12.60)	9.34 (6.10–14.50)	0.112
TNFRII	5.66 (3.63–14.80)	6.86 (4.06–13.90)	0.147
GITR	0.36 (0.12–1.39)	0.51 (0.07–1.89)	0.082
LAG-3	0.09 (0.03–0.18)	0.09 (0.03–1.46)	0.826
LAP-TGF-β	3.96 (1.96–14.80)	4.38 (2.35–15.8)	0.713
OX-40	9.22 (5.35–14.60)	7.92 (5.38–14.10)	0.809
CD69	4.21 (0.97–23.15)	5.62 (1.34–28.60)	0.606

Data are presented as medians (interquartile ranges) and were compared with the Mann-Whitney *U* test.

* indicates a significant difference after controlling for a false discovery rate (*q*) set at 0.10 (number of comparisons *m* = 11).

**Fig 2:**
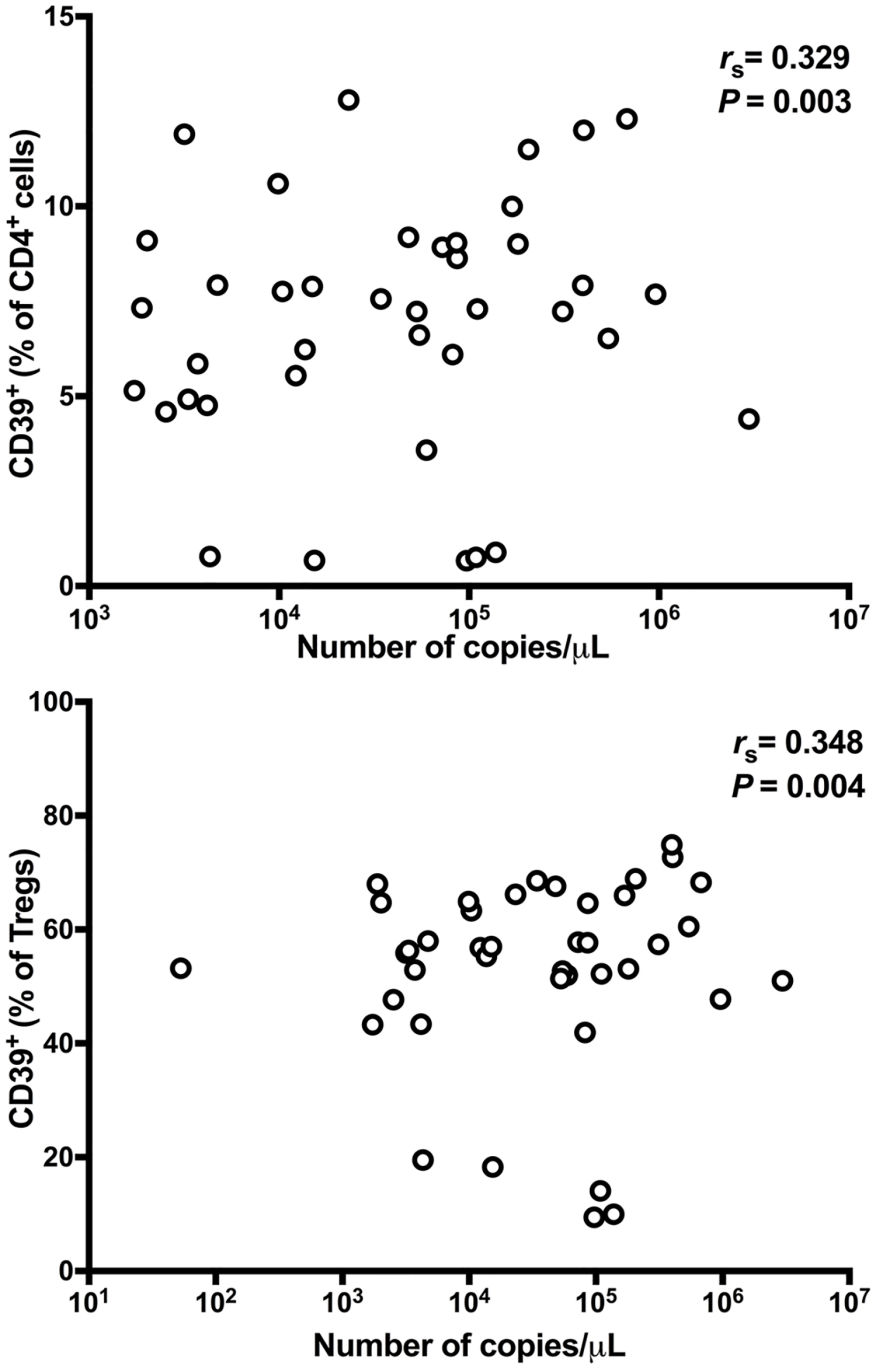
The expansion of CD4^+^CD39^+^ T-cell populations in microfilaremics is directly proportional to their microfilarial load. The frequencies of CD4^+^CD39^+^ T cells (% of all CD4^+^ T cells that express CD39; upper panel) and CD39^+^ Treg cells (% of all CD4^+^CD25^hi^CD127^-^FoxP3^+^ T cells that express CD39; lower panel) were plotted against the number of *Mansonella ozzardi*-specific ITS-2 amplicon copies obtained by quantitative real-time PCR, a proxy of microfilarial load. Data analyzed for 48 subjects using the Spearman rank correlation test.

We next examined the classical Treg cell compartment, phenotypically defined as CD4^+^CD25^hi^CD127^-^FoxP3^+^ cells, using the gating strategies shown in S4 and S5 Figs. Treg cells comprised identical proportions (median, 1.5%) of CD4^+^ T cells circulating in Fil+ and Fil- subjects, indicating that *M*. *ozzardi* infection did not induce an expansion of the Treg cell compartment. However, the analysis of surface marker expression on Treg cells revealed a significantly greater proportion of CD39^+^ Tregs in microfilaremics compared to uninfected controls (median, 69.7% vs. 60.9%). CD39^+^ Treg cells accounted for a median of 1.1% and 0.9% of all CD4^+^ T cells circulating in infected and uninfected individuals, respectively. Therefore, ~15–16% of CD39^+^ cells within the CD4^+^ compartment were classical Treg cells. No significant difference was found in the frequencies of Treg cells expressing all other molecules listed in Table 3, except for the CD39^-^PD-1^+^ subset (increased in Fil+ subjects). Overall, similar results were obtained when comparing IgG_4_H and IgG_4_L subjects (S11 Table). Interestingly, CD39^+^ Treg cells from microfilaremics and uninfected controls more often co-express a wide range of other regulatory molecules (CTLA-4, LAP-TGF-β, LAG-3, TNFRII, GITR, and OX-40) and activation markers (HLA-DR and CD69) than do CD39^-^ Treg cells (S6 Fig). These findings are consistent with the notion that CD39 expression characterizes a functionally more “suppressive” Treg subtype [32]. Because the frequencies of CD39^+^ CD4^+^ T cells and CD39^+^ classical Treg cells correlated positively with the number of *M*. *ozzardi*-specific ITS-2 amplicon copies in microfilaremics (Fig 2), we conclude that the *M*. *ozzardi*-associated expansion of CD39^+^ T-cell populations is directly proportional to the microfilarial load.

**Table 3:** Frequency of CD4^+^CD25^hi^CD127^-^FoxP3^+^ Treg cells expressing regulation and activation markers in microfilaremic subjects (Fil+) and uninfected controls (Fil-).

Marker	Value for group (% of Treg cells)		*P* value
	Fil-	Fil+	
No. of subjects	33	48	
CD69	3.51 (1.68–9.60)	3.32 (1.69–8.23)	0.592
CTLA-4	0.49 (0.23–6.24)	0.69 (0.42–7.21)	0.417
Intracellular CTLA-4	78.60 (62.20–83.05)	77.00 (68.90–78.60)	0.673
HLA-DR	26.75 (8.31–45.67)	28.95 (14.30–55.20)	0.494
PD-1	3.37 (1.76–9.50)	4.50 (1.98–10.30)	0.132
TNFRII	45.40 (28,98–57.90)	42.25 (27.00–51.30)	0.972
GITR	0.33 (0.12–1.16)	0.43 (0.18–1.45)	0.161
LAG-3	0.13 (0.05–0.98)	0.17 (0.05–1.07)	0.173
LAP-TGF-β	7.08 (3.84–16.98)	6.21(3.92–14.80)	0.912
OX-40	3.62 (1.59–12.56)	3.68 (1.61–13.60)	0.784
CD39	60.90 (48.05–62.40)	69.70 (62.3–70.12)	0.009*
CD39^+^CD69^+^	3.31 (0.98–8.90)	2.84 (0.73–12.50)	0.286
CD39^+^CTLA-4^+^	0.55 (0.10–14.70)	0.91 (0.28–25.40)	0.967
CD39^+^intracellular CTLA-4^+^	84.80 (34.56–89.60)	83.00 (68.40–91.30)	0.351
CD39^+^HLA-DR^+^	33.80 (12.39–66.70)	33.05 (16.40–64.30)	0.855
CD39^+^PD-1^+^	3.55 (1.39–15.20)	4.19 (1.61–14.30)	0.323
CD39^+^TNFRII^+^	48.85 (24.62–74.88)	44.75 (36.10–69.72)	0.516
CD39^+^GITR^+^	0.35 (0.06–1.30)	0.44 (0.07–2.10)	0.416
CD39^+^LAG-3^+^	0.00 (0.00–3.94)	0.19 (0.00–1.18)	0.077
CD39^+^LAP-TGF-β ^+^	7.71 (3.52–9.40)	6.18 (2.71–17.10)	0.681
CD39^+^OX-40^+^	4.53 (2.32–20.15)	4.50 (1.71–18.30)	0.891
CD39^-^CD69^+^	3.34 (0.68–12.98)	2.47 (0.30–13.71)	0.210
CD39^-^CTLA-4^+^	0.83 (0.08–4.50)	0.98 (0.04–6.30)	0.897
CD39^-^intracellular CTLA-4^+^	61.4 (38.12–67.60)	65.50 (37.60–72.70)	0.176
CD39^-^HLADR^+^	12.35 (6.32–38.40)	13.40 (8.16–37.20)	0.435
CD39^-^PD-1^+^	4.43 (0.87–12.70)	7.79 (2.98–16.75)	0.001*
CD39^-^TNFRII^+^	31.3 (8.45–46.72)	33.05 (15.40–47.50)	0.414
CD39^-^GITR^+^	0.14 (0.02–1.70)	0.11 (0.01–1.58)	0.520
CD39^-^LAG-3^+^	0.00 (0.00–1.20)	0.00 (0.00–1.78)	0.041
CD39^-^LAP-TGF-β^+^	4.90 (1.05–12.20)	2.65 (0.62–11.73)	0.895
CD39^-^OX-40^+^	2.36 (0.08–6.50)	2.93 (0.07–7.12)	0.645

Data are presented as medians (interquartile ranges) and were compared with the Mann-Whitney *U* test.

* indicates significant differences after controlling for a false discovery rate (*q*) set at 0.10 (number of comparisons *m* = 31).

### CD39 blocking *in vitro* inhibits lymphocyte proliferation and alters cytokine responses after antigen stimulation

**Fig 3:**
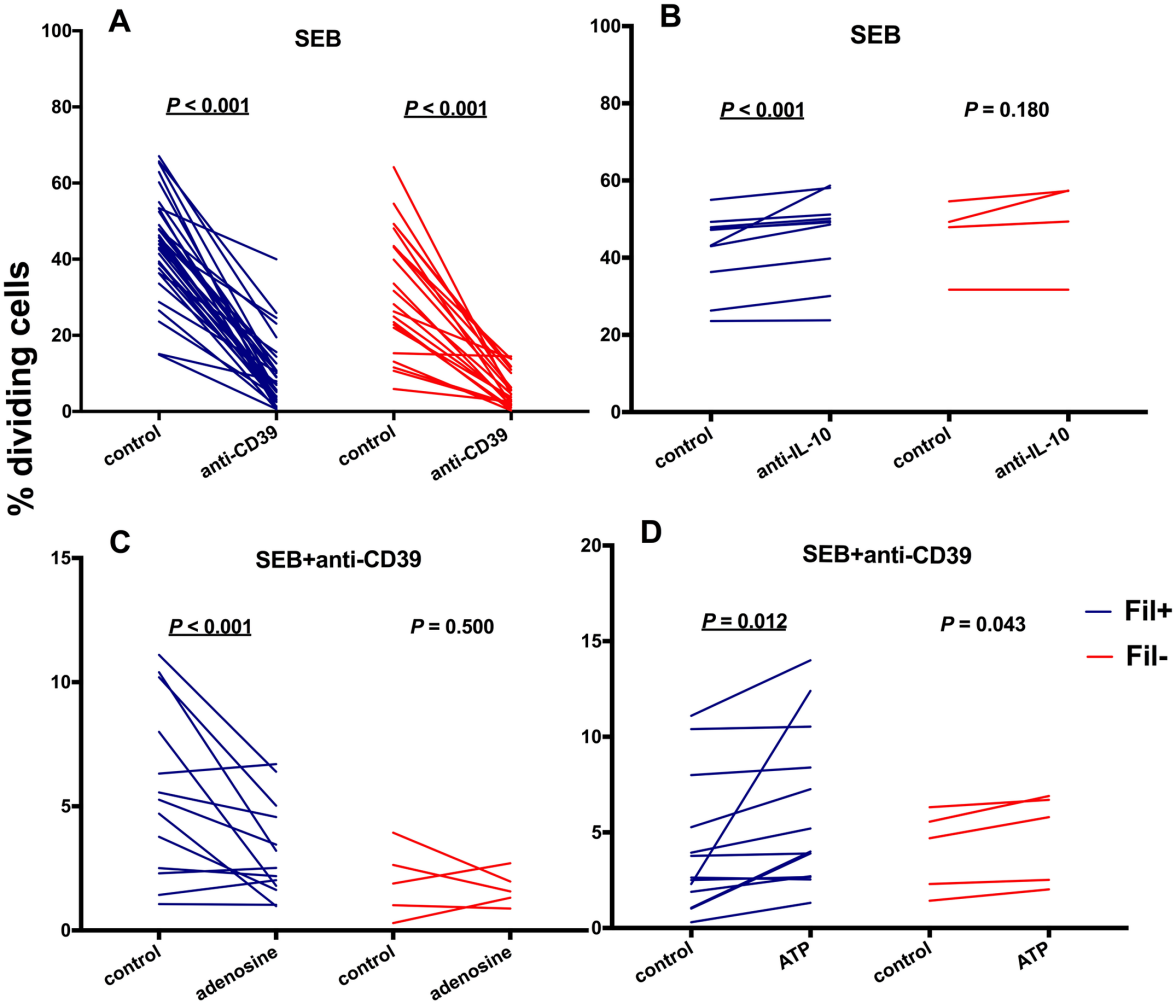
CD39 blocking markedly inhibits antigen-driven lymphocyte proliferation. PBMC from microfilaremic (Fil+) and uninfected (Fil-) subjects were labeled with CellTrace Violet and incubated with *Staphylococcus aureus* enterotoxin B (SEB). The net % of divided cells was compared in the presence or absence of anti-CD39 antibody (“anti-CD39” and “control”, respectively; panel A), in the presence or absence of anti-IL-10 antibody (“anti-IL-10” and “control”, respectively; panel B), in the presence of anti-CD39 antibody with or without 2 mM adenosine added to the culture medium (“adenosine” and “control”, respectively; panel C), and in the presence of anti-CD39 antibody with or without 2 mM ATP added to the culture medium (“ATP” and “control”, respectively; panel D). Individual data are presented for 36 Fil+ and 21 Fil- subjects (panel A), 10 Fil+ and 4 Fil- subjects (panel B) or 13 Fil+ and 5 Fil- subjects (panels C and D); they were compared using the Wilcoxon signed rank test. Significant *P* values after controlling for a false discovery rate (*q*) set at 0.10 are underlined (number of comparisons *m* = 4 for each group, Fil+ and Fil-).

To explore the immunomodulatory consequences of surface expression of CD39 in circulating CD4^+^ T cells, we used an anti-CD39 antibody to neutralize this molecule [30]. We observed that SEB-driven lymphocyte proliferation *decreased* significantly in the presence of CD39-blocking antibody (Fig 3A), although proliferation increased, as expected [2], in the presence of neutralizing anti-IL-10 antibody (Fig 3B). SEB-induced lymphocyte proliferation in the presence of anti-CD39 antibody was further inhibited by adding 2mM adenosine to the culture medium (Fig 3C), being partially restored by adding 2mM ATP (Fig 3D). Interestingly, comparable inhibitory effects of CD39 blocking on cell proliferation *in vitro* were observed in Fil+ and Fil- subjects.

**Fig 4:**
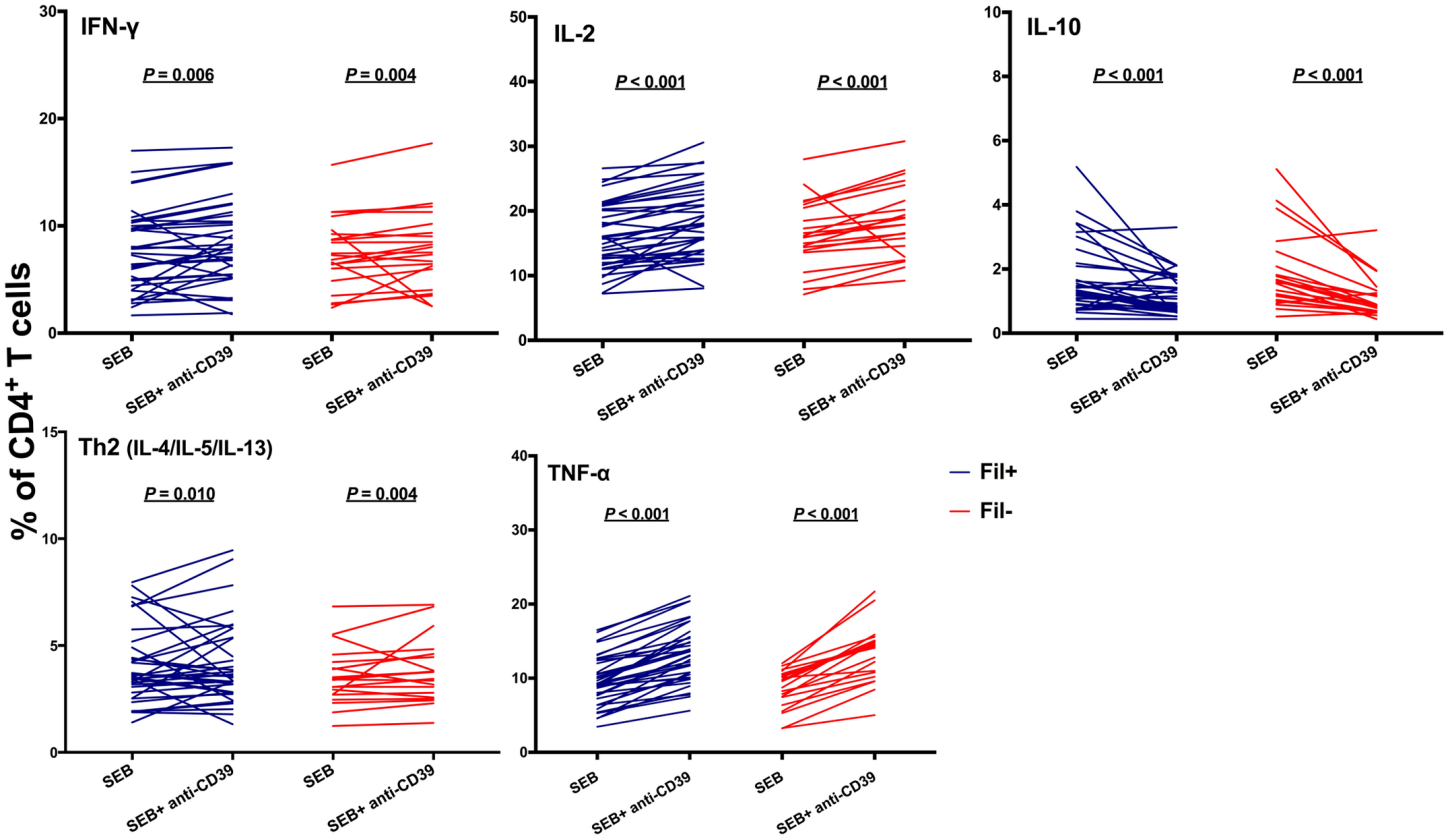
CD39 blocking alters antigen-driven cytokine production by CD4^+^ T cells. PBMC from microfilaremic (Fil+) and uninfected (Fil-) subjects were stimulated with *Staphylococcus aureus* enterotoxin B (SEB) in the presence or absence of anti-CD39 antibody and stained for intracellular cytokines. The % of CD4^+^ T cells producing each cytokine was estimated by flow cytometry. Individual data are presented for 40 Fil+ and 28 Fil- subjects and were compared using the Wilcoxon signed rank test. Significant *P* values after controlling for a false discovery rate (*q*) set at 0.10 are underlined (number of comparisons *m* = 5 for each group, Fil+ and Fil-).

**Fig 5:**
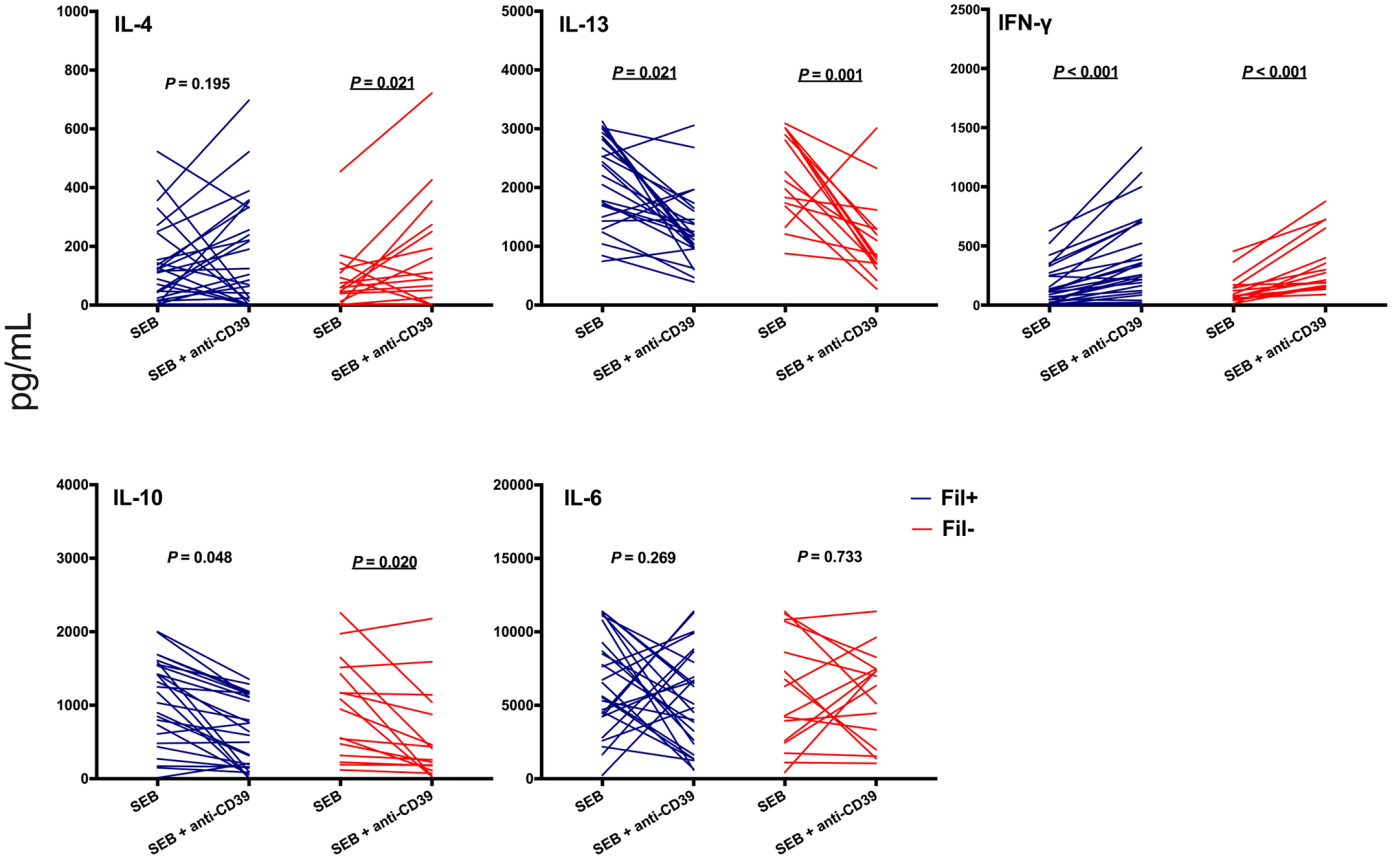
CD39 blocking alters antigen-driven cytokine secretion in PBMC culture supernatants. PBMC from microfilaremic (Fil+) and uninfected (Fil-) subjects were stimulated with *Staphylococcus aureus* enterotoxin B (SEB) in the presence or absence of anti-CD39 antibody. After 72 h of culture, supernatants were harvested and assessed for cytokines. Data are presented as individual cytokine concentrations (pg/mL) for 27 Fil+ and 15 Fil- subjects and were compared using the Wilcoxon signed rank test. Significant *P* values after controlling for a false discovery rate (*q*) set at 0.10 are underlined (number of comparisons *m* = 5 for each group, Fil+ and Fil-).

Antibody-mediated CD39 neutralization also exerted profound effects on SEB-driven cytokine production. We observed increased proportions of CD4^+^ T cells producing IFN-γ, IL-2, TNF-α, and Th2-type cytokines, but a decreased proportion of CD4^+^ T cells producing IL-10, in the presence of anti-CD39 antibody (Fig 4). These changes were reversed in the presence of 2mM adenosine (S7 Fig). Accordingly, levels of IL-4, and IFN-γ in culture supernatants increased, while those of IL-13, and IL-10 significantly decreased, following CD39 blocking *in vitro* (Fig 5). CD39 blocking caused similar changes in SEB-driven cytokine responses in Fil+ and Fil- subjects (Figs 4 and 5), although not all comparisons reached statistical significance due to the relatively small sample sizes.

### Increased Ki67 expression in circulating CD39^+^ CD4^+^ T cells and Treg cells is reversed by CD39 blocking

**Fig 6:**
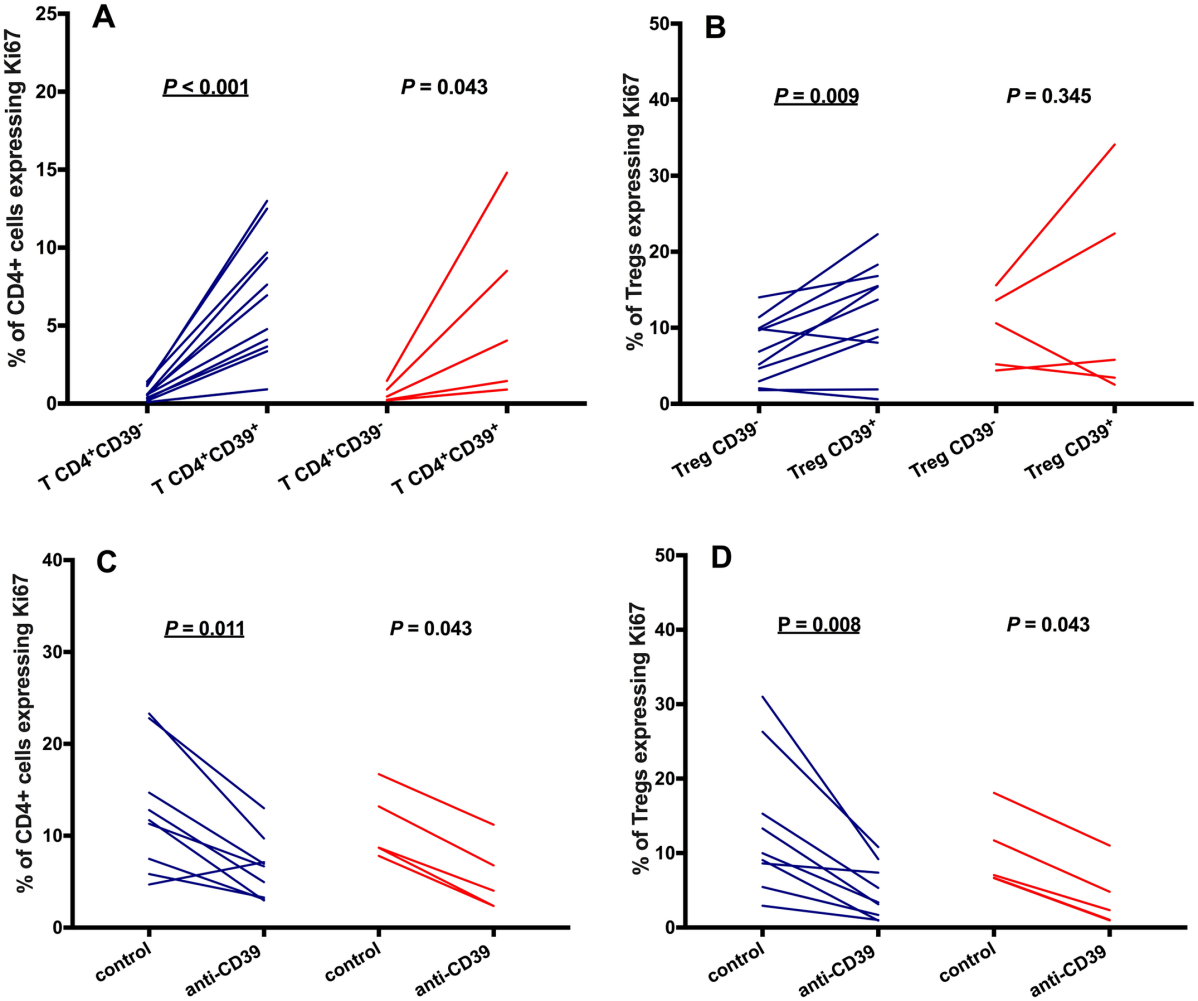
Reduced Ki67 expression in CD4^+^CD39^+^ T cells following CD39 blocking. PBMC from microfilaremic (Fil+) and uninfected (Fil-) subjects were stained for intracellular Ki67, an indicator of T-cell proliferation. We first compared the proportion of Ki67^+^ cells among CD4^+^CD39^+^ and CD4^+^CD39^-^ T cells (panel A) and CD39^+^ and CD39^-^ Treg (CD4^+^CD25^hi^CD127^-^FoxP3^+^) cells (panel B). Next, we compared the proportion of Ki67^+^ cells among CD4^+^ T cells (panel C) and Treg cells (panel D) in the presence or absence of anti-CD39 antibody (“anti-CD39” and “control”, respectively). Individual data are shown for 11 Fil+ and 5 Fil- subjects (panels A and B) or 9 Fil+ and 5 Fil- subjects (panels C and D) and were compared using the Wilcoxon signed rank test. Significant *P* values after controlling for a false discovery rate (q) set at 0.10 are underlined (number of comparisons *m* = 5 for each group, Fil+ and Fil-).

Intracellular Ki67 expression was measured to explore the effects of CD39 on T-cell proliferation. We first observed an increased expression of Ki67 by CD39^+^ CD4^+^ T cells, compared to their CD39^-^ counterparts. This change was statistically significant in microfilaremics but not in uninfected controls, although the trends were similar (Fig 6A). We also found an increased expression of Ki67 in the CD39^+^ subset of Treg cells, compared to CD39^-^ Treg cells, in microfilaremics (Fig 6B). These and previous findings [32] indicate that CD39 expression delineates CD4^+^ T cell subsets with enhanced proliferative ability. Indeed, CD39 blocking significantly reduced the proportion of Ki67^+^ CD39^+^ CD4^+^ T cells (Fig 6C) and Ki67^+^ CD39^+^ Treg cells (Fig 6D). The trends were similar regardless of the infection status, but only reached statistical significance among microfilaremics. This further confirms the suppressive effect of CD39 neutralization on T-cell proliferation.

## Discussion

The filarial nematode *M*. *ozzardi* challenges the common view that chronic helminth infections will necessarily elicit immunomodulatory responses such as those thoroughly described in schistosomiasis, lymphatic filariasis, and intestinal nematode infections [1,3,4]. We observed decreased plasma levels of some inflammatory cytokines in microfilaremics, compared to uninfected controls living in the same communities, but Fil+ and Fil- groups had similar proportions of CD4^+^ T cells producing IFN-γ, IL-2, IL-10, TNF-α, and Th2-type cytokines, as well as similar levels of secreted IL-6, IL-13, IL-10, IL-4, and IFN-γ in culture supernatants, following antigen stimulation. A concomitant increase in plasma concentrations of both inflammatory (IL-6) and regulatory (IL-10) cytokines was recently described in *M*. *ozzardi* infections in Brazil [33], but further comparisons with our data are limited by the lack of concurrent analyses of antigen-driven cytokine production *in vitro* [33].

Moreover, T-cell responses to filarial and unrelated antigens are not attenuated in subjects harboring *M*. *ozzardi* microfilariae. Our data suggest that *M*. *ozzardi* infection, which is highly prevalent in riverine communities across the Amazon Basin [8], is unlikely to suppress T-cell responses to co-occurring pathogens, such as malaria parasites, in endemic populations.

How Treg cells exert their suppressive effects remains incompletely understood, but CD39 is thought to play a crucial immunoregulatory role in these cells [15]. Indeed, surface expression of CD39 appears to confer enhanced proliferative and suppressive ability to induced Treg cells [32,34]. Moreover, CD39 boosts the differentiation of type 1 regulatory (Tr1) cells, characterized by the production of IL-10 and lack of FoxP3 expression, which are able to limit inflammation and favor immune tolerance [34]. CD39 hydrolyses extracellular ATP (eATP) and ADP into AMP, while another ectonucleotidase, CD73, dephosphorylates AMP to adenosine. eATP activates P2 purignergic and pyrimidinergic receptors expressed by T cells, B cells, dendritic cells, macrophages, and neutrophils, triggering a range of proinflammatory responses. Adenosine, in contrast, is a labile molecule that binds to the adenosine receptor 2A expressed on effector T cells and dampens cell proliferation and inflammatory cytokine secretion [15]. Adenosine concentrations are further regulated by adenosine deaminase (ADA), which catalyzes the deamination of adenosine generating inosine and ammonia. Human ADA1 binds CD26 on T-cells, favoring adenosine turn-over [35]. Therefore, the interplay of CD39 with CD73 and CD26 regulates the levels of eATP, ADP, AMP, and adenosine, with major consequences for the purinergic control of inflammation and adaptative immune responses [34].

Increased expression of CD39 by circulating Treg cells has been associated with disease progression in tuberculosis [36] and HIV/AIDS [37–39]. Moreover, CD39 is overexpressed by several types of cancer cells, in addition to tumor-infiltrating T cells, suggesting that CD39 and purinergic signaling can directly modulate tumor growth, metastasis and angiogenesis, in addition to inducing immune tolerance, further favoring cancer progression [40]. Indeed, blocking CD39 *in vivo* with specific antagonists or monoclonal antibodies currently in preclinical development has been suggested as an immunomodulatory intervention to enhance effector T-cell responses in HIV infection [37,38] and in cancer [40].

However, the pool of circulating CD4^+^ T cells expressing CD39 comprises not only Treg cells with enhanced co-expression of regulatory markers and greater suppressive ability. In fact, 5–7% of all circulating CD4^+^ T cells expressed CD39 in our study subjects, and CD39^+^ Treg cells represented only 15–16% of the pool of circulating CD39^+^CD4^+^ T cells in microfilaremic and uninfected study subjects. Moreover, CD39 is constitutively expressed by the vast majority (>90%) of B cells and monocytes and by small proportions (5% or less) of CD8^+^ T cells [40]. A particularly interesting subset of CD39^+^CD4^+^ T cells comprises the partially characterized CD25^-^FoxP3^-^ “inducer” T (Tind) cells that *promote* and *potentiate*, rather than suppress, T-cell proliferation and inflammatory cytokine production [41,42]. Tind cells appear to be functionally similar to newly described activated effector CD4^+^ T cells that co-express CD39 and CD26, have no suppressive effect, and are prone to apoptosis [43]. Co-culture with Tind cells enhances the proliferation of CD4^+^CD25^-^CD39^-^ “responder” T cells (Tres) from healthy donors, an effect that can be partially reversed by anti-CD39 monoclonal antibodies [41]. Moreover, co-culture of Tres with Tind cells enhances the production of IFN-γ, TNF-α, GM-CSF, IL-6, and IL-10 [41]. These findings imply that increased CD39 expression and regulatory ability are not necessarily coupled in all circulating CD4^+^ T cell subsets. The dramatic decrease in antigen-driven cell proliferation observed in both microfilaremic and uninfected individuals in the presence of CD39-blocking antibody (Fig 3A) is consistent with a predominance of Tind cells in the CD39^+^CD4^+^ pool of circulating T cells from our study population. Therefore, the relative proportions of CD39^+^ Treg and Tind cells may determine the patterns of immune homeostasis observed in human populations exposed to different environmental antigens and pathogens. In addition, this balance may determine the outcome of immunomodulatory interventions based on CD39 blocking.

The main limitation of this study is its cross-sectional design. Therefore, we were able to identify statistically significant associations between current infection and the proportion of certain CD4^+^ T cell populations circulating in the peripheral blood, but our study design does not allow us to infer causal relationships. Moreover, phenotypic characterization of CD4^+^ T cells is restricted to the circulating compartment, which may not be representative of the T cell population in infected subjects. Analyses of post-treatment samples could theoretically help to delineate infection-related changes in T-cell responses that can be reversed in the absence of helminth-derived antigenic stimulation. However, given that ivermectin, the first-line treatment for *M*. *ozzardi* infection, is highly efficacious against microfilariae but does not appear to kill adult worms [8,19], PBMC samples collected after treatment may still be stimulated by circulating excreted-secreted soluble antigens that are chronically released by adult worms.

In conclusion, it is tempting to speculate that the balance between suppressive CD4^+^CD39^+^ Treg cells and immunostimulatory CD4^+^CD25^-^FoxP3^-^CD39^+^ Tind cells is a key factor contributing to the unexpected patterns of immune regulation found in *M*. *ozzardi* infection. We suggest that an increased frequency of Tind cells in our microfilaremics might have prevented lymphocyte hyporesponsiveness despite the concomitant expansion of the CD4^+^CD39^+^ Treg subset. However, the CD39^+^ cell pool remains uncharacterized in other chronic helminth infections that induce typical immunomodulatory responses. Therefore, further studies on CD39^+^ cells may help to unveil some of the biochemical and molecular pathways whereby different helminths manipulate their hosts’ immunity.

## Supporting information

S1 Fig Map showing the geographic location of the six riverine villages along Purus River, municipality of Boca do Acre, northwestern Brazil. MV = Monte Verde, VP = Valparaíso, BV = Boa Vista, RT = Retiro, NV = Nova Vida, and SP = Sao Pedro. The location of the town of Boca do Acre is also shown.
(DOCX)

S2 Fig Gating strategy to define CD4^+^ T cell subpopulations (co)expressing HLA-DR, CD69, TNFRII, PD-1 and CTLA-4. A, Time; B, Singlets; C, Lymphocytes were selected for their size and complexity; D, Selection of viable cells; E, Selection of CD3^+^ cells; F, Selection of CD4^+^ T cells. Expression of TNFRII (G) PD-1 (H), CD69 (I), CTLA-4 (J), and HLA-DR (L) was measured as shown.
(DOCX)

S3 Fig Gating strategy to define CD4^+^ T cell subpopulations (co)expressing intracellular CTLA-4, OX-40, TGF-β-LAP, GITR, and LAG-3. A, Time; B, Singlets; C, Lymphocytes were selected for their size and complexity; D, Selection of viable cells; E, Selection of CD3^+^ cells; F, Selection of CD4^+^ T cells. Expression of intracelular CD39 (G), CTLA-4 (H), OX-40 (I), LAP-TGF-β (J), GITR (L), and LAG-3 (M) was evaluated as shown.
(DOCX)

S4 Fig Gating strategy to define CD4^+^ T cell subpopulations (co)expressing CD39 and FOXP3 and HLA-DR, CD69, TNFRII, PD-1, and CTLA-4. A, Time; B, Singlets; C, Lymphocytes were selected for their size and complexity; D, Selection of viable cells; E, Selection of CD3^+^ cells; F, Dual labelling for CD4 and CD25 to define CD4^+^CD25^+^ cells; G, Selection of CD4^+^CD25^+^ cells that do not express CD127; H, Selection, from the CD25^+^CD4^+^CD127^-^ population, of lymphocytes co-expressing FOXP3 and CD39; H1, CD4^+^CD25^+^CD127^-^CD39^+^FOXP3^-^ T cells; H2, CD4^+^CD25^-^CD127^+^CD39^+^FOXP3^+^ T cells; H3, CD4^+^CD25^+^CD127^-^CD39^-^FOXP3^+^ T cells. Expression of TNFRII (I) PD-1 (J), CD69 (L), CTLA-4 (M), and HLA-DR (N) was evaluated as shown.
(DOCX)

S5 Fig Gating strategy to define CD4^+^ T cell subpopulations (co)expressing CD39, FOXP3 and intracellular CTLA-4, OX-40, TGF-β-LAP, GITR and LAG-3. A, Time; B, Singlets; C, Lymphocytes were selected for their size and complexity; D, Selection of viable cells; E, Selection of CD3^+^ cells; F, Dual labelling for CD4 and CD25 to define CD4^+^CD25^+^ cells; G, Selection of CD4^+^CD25^+^ cells that do not express CD127; H, Selection, from the CD25^+^CD4^+^CD127^-^ population, of lymphocytes co-expressing FOXP3 and CD39; H1, CD4^+^CD25^+^CD127^-^CD39^+^FOXP3^-^ T cells; H2, CD4^+^CD25^-^CD127^+^CD39^+^FOXP3^+^ T cells; H3, CD4^+^CD25^+^CD127^-^CD39^-^FOXP3^+^ T cells. Expression of intracellular CTLA-4 (I), OX-40 (J), LAP-TGF-β (L), GITR (M), and LAG-3 (N) was evaluated as shown.
(DOCX)

S6 Fig CD39^+^ Treg cells from microfilaremics and uninfected controls more often express intracellular CTLA-4, LAP-TGB-β, LAG-3, TNFRII, GITR, OX-40, HLA-DR, and CD69 (but not PD-1) than CD39^-^ Treg cells. We compared the frequencies of CD39^+^ and CD39^-^ Treg cells (defined as CD4^+^CD25^hi^CD127^-^FoxP3^+^ T cells) that expressed a range of regulatory and activation markers. Data are shown for 48 Fil+ and 33 Fil- subjects and were compared using the Wilcoxon signed rank test. Only significant *P* values after controlling for a false discovery rate (*q*) set at 0.10 (*m* = 9) are shown.
(DOCX)

S7 Fig Changes in the proportions of CD4^+^ T cells producing IFN-γ, IL-2, TNF-α, Th2-type cytokines, and IL-10 in the presence of anti-CD39 antibody are reversed by adding 2mM adenosine. PBMC from microfilaremic (Fil+) and uninfected (Fil-) subjects were stimulated with *Staphylococcus aureus* enterotoxin B (SEB) in the presence or absence of anti-CD39 antibody, stained for intracellular cytokines, and then incubated with 2mM adenosine. The % of CD4^+^ T cells producing each cytokine was estimated by flow cytometry. Data are presented for 11 Fil+ and 5 Fil- subjects and were compared using the Wilcoxon signed rank test. Only significant *P* values after controlling for a false discovery rate (*q*) set at 0.10 (*m* = 5 for each group [Fil+ and Fil-] and each pair of experimental conditions [SEB vs. SEB+anti-CD39, SEB vs. SEB+anti-CD39+adenosine; SEB+anti-CD39 vs. SEB+anti-CD39+adenosine]) are shown.
(DOCX)

S1 Table Panel 1: Monoclonal antibodies used to characterize regulatory and activation markers on CD4 ^+^ T cells. (PDF)

S2 Table Panel 2: Monoclonal antibodies used to characterize regulatory and activation markers on CD4 ^+^ T cells. (PDF)

S3 Table Panel 3: Monoclonal antibodies used to characterize Ki67-expressing Treg cells. (PDF)

S4 Table Panel 4: Monoclonal antibodies used for intracellular cytokine staining in CD4 ^+^ T cells. (PDF)

S5 Table Frequency of clinical signs and symptoms reported by *M*. *ozzardi*-infected subjects and uninfected controls. (PDF)

S6 Table Demographic, hematologic, and clinical characteristics of study participants according to levels of BmA-specific IgG_4_ antibodies. Subjects with BmA antibody levels above the overall median were defined as IgG4H, while those with BmA antibody levels below the overall median were defined as IgG4L.
(PDF)

S7 Table Levels of plasma cytokines in microfilaremic subjects (Fil+) and uninfected controls (Fil-). (PDF)

S8 Table Proportion of CD4^+^ T cells from microfilaremic subjects (Fil+) and uninfected controls (Fil-) producing specific cytokines upon stimulation *in vitro* with filarial (BmA) and unrelated (SEB) antigen. (PDF)

S9 Table Levels of cytokines in PBMC culture supernatants from microfilaremic subjects (Fil+) and uninfected controls (Fil-) after stimulation *in vitro* with filarial (BmA) and unrelated (SEB) antigen. (PDF)

S10 Table Frequency (%) of T CD4^+^ lymphocytes expressing regulation and activation markers in study participants divided into IgG4L and IgGH groups according to their levels of BmA-specific IgG_4_ antibodies. (PDF)

S11 Table Frequency (%) of CD4^+^CD25^hi^CD127^-^FoxP3^+^ (Treg) cells expressing regulatory and activation markers in study participants divided into IgG4L and IgGH groups according to their levels of BmA-specific IgG_4_ antibodies. (PDF)
