# Supplementary material for: CD39 and immune regulation in a chronic helminth infection: The puzzling case of Mansonella ozzardi
Source: PLoS Negl Trop Dis. 2018 Mar 5;12(3):e0006327. doi: 10.1371/journal.pntd.0006327 (PMC5854421; doi:10.1371/journal.pntd.0006327)
Supplement: S1 Fig — MV = Monte Verde, VP = Valparaíso, BV = Boa Vista, RT = Retiro, NV = Nova Vida, and SP = Sao Pedro. The location of the town of Boca do Acre is also shown. (DOCX) [file pntd.0006327.s001.docx]

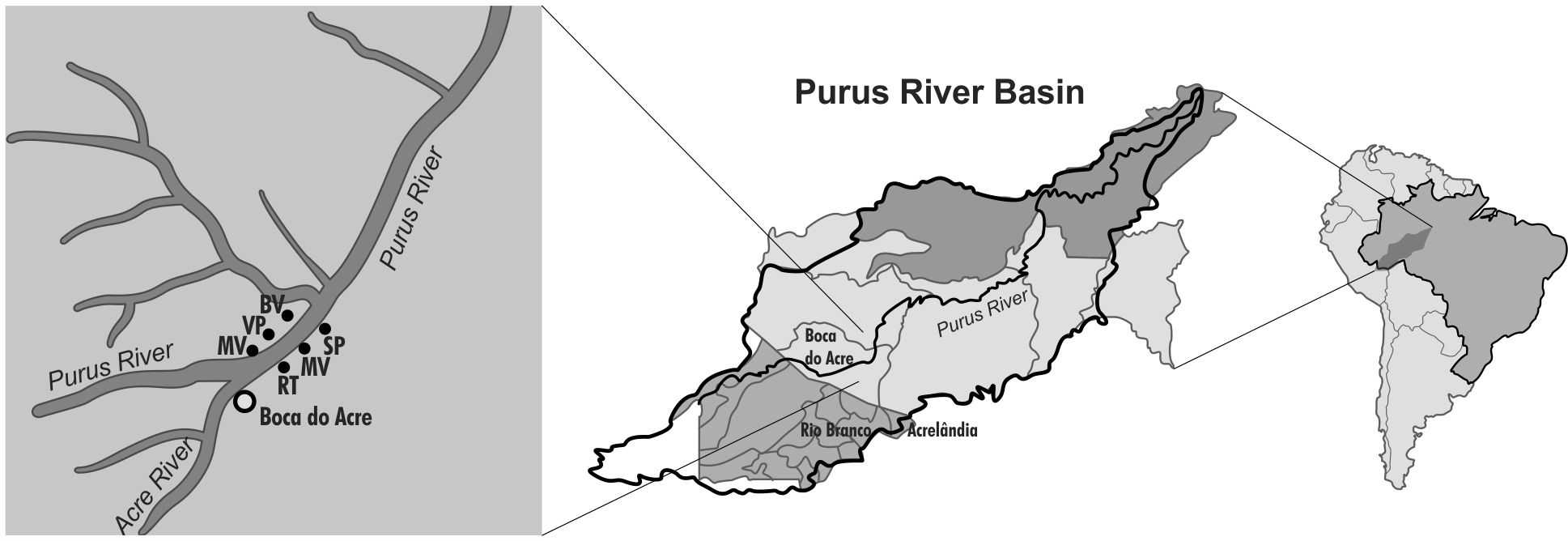


**S1 Fig.** **Map showing the geographic location of the six riverine villages along Purus River, municipality of Boca do Acre, northwestern Brazil.** MV = Monte Verde, VP = Valparaíso, BV = Boa Vista, RT = Retiro, NV = Nova Vida, and SP = Sao Pedro. The location of the town of Boca do Acre is also shown.
