# Supplementary material for: CD39 and immune regulation in a chronic helminth infection: The puzzling case of Mansonella ozzardi
Source: PLoS Negl Trop Dis. 2018 Mar 5;12(3):e0006327. doi: 10.1371/journal.pntd.0006327 (PMC5854421; doi:10.1371/journal.pntd.0006327)
Supplement: S2 Fig — A, Time; B, Singlets; C, Lymphocytes were selected for their size and complexity; D, Selection of viable cells; E, Selection of CD3+ cells; F, Selection of CD4+ T cells. Expression of TNFRII (G) PD-1 (H), CD69 (I), CTLA-4 (J), and HLA-DR (L) was measured as shown. (DOCX) [file pntd.0006327.s002.docx]

**
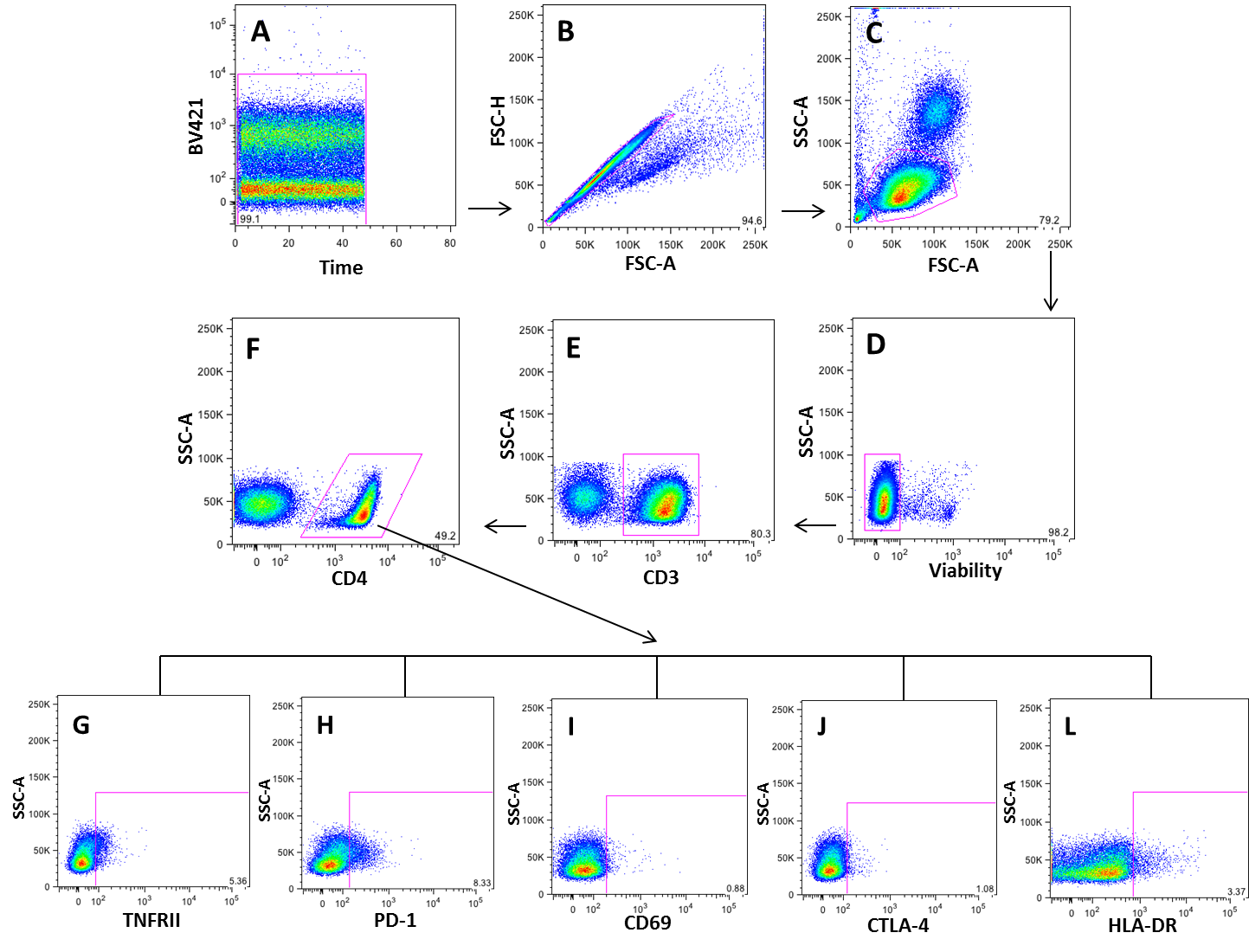
**

**S2 Fig. Gating strategy to define CD4^+^ T cell subpopulations (co)expressing HLA-DR, CD69, TNFRII, PD-1 and CTLA-4.** A, Time; B, Singlets; C, Lymphocytes were selected for their size and complexity; D, Selection of viable cells; E, Selection of CD3^+^ cells; F, Selection of CD4^+^ T cells. Expression of TNFRII (G) PD-1 (H), CD69 (I), CTLA-4 (J), and HLA-DR (L) was measured as shown.
