# Supplementary material for: CD39 and immune regulation in a chronic helminth infection: The puzzling case of Mansonella ozzardi
Source: PLoS Negl Trop Dis. 2018 Mar 5;12(3):e0006327. doi: 10.1371/journal.pntd.0006327 (PMC5854421; doi:10.1371/journal.pntd.0006327)
Supplement: S3 Fig — A, Time; B, Singlets; C, Lymphocytes were selected for their size and complexity; D, Selection of viable cells; E, Selection of CD3+ cells; F, Selection of CD4+ T cells. Expression of intracelular CD39 (G), CTLA-4 (H), OX-40 (I), LAP-TGF-β (J), GITR (L), and LAG-3 (M) was evaluated as shown. (DOCX) [file pntd.0006327.s003.docx]

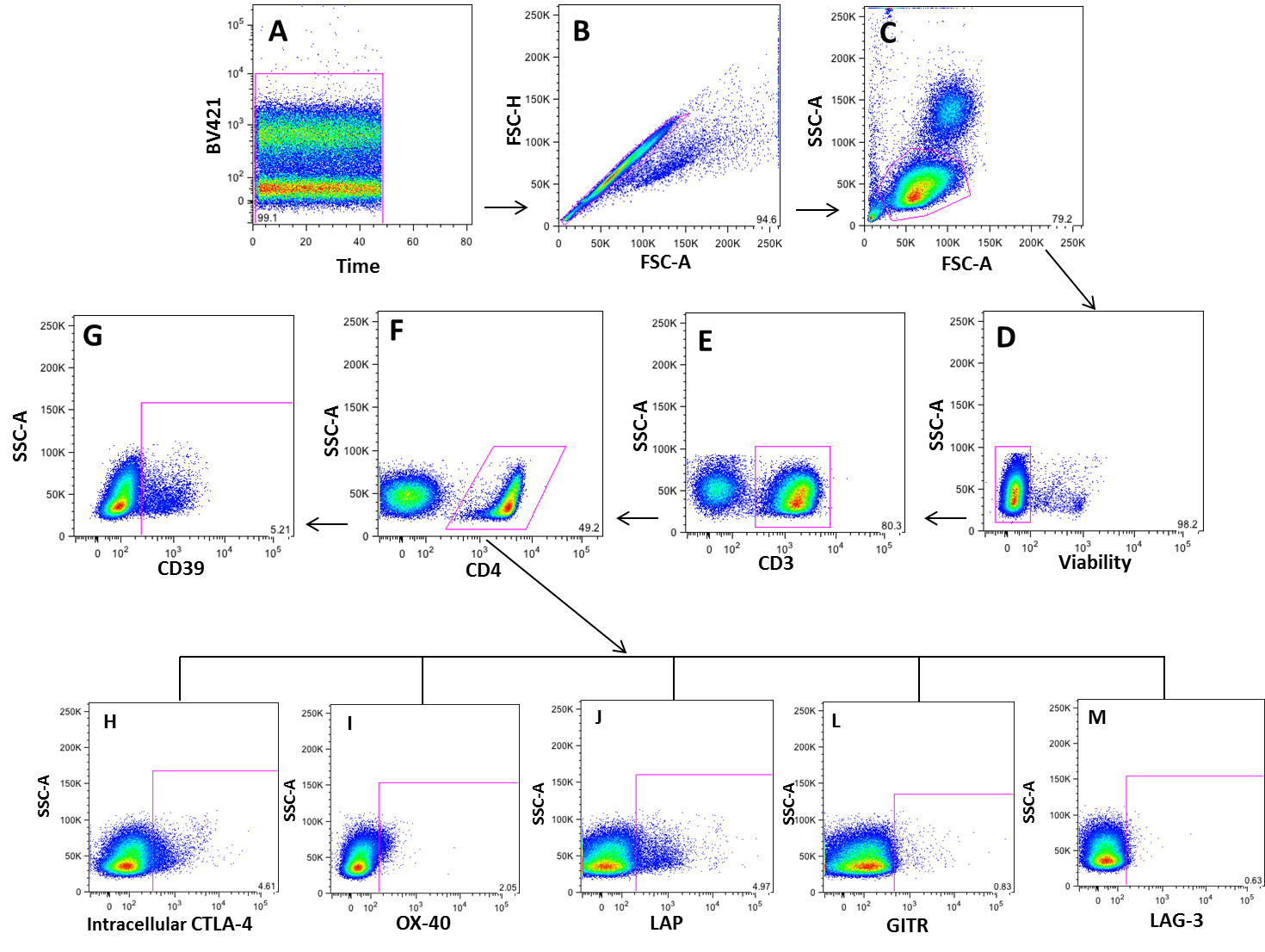


**S3 Fig.** **Gating strategy to define CD4^+^ T cell subpopulations (co)expressing intracellular CTLA-4, OX-40, TGF-β-LAP, GITR and HLA-DR.** A, Time; B, Singlets; C, Lymphocytes were selected for their size and complexity; D, Selection of viable cells; E, Selection of CD3^+^ cells; F, Selection of CD4^+^ T cells. Expression of intracelular CD39(G) CTLA-4 (H) OX-40 (I), LAP-TGF-β (J), GITR (L) and HLA-DR (M) was evaluated as shown.
