# Supplementary material for: CD39 and immune regulation in a chronic helminth infection: The puzzling case of Mansonella ozzardi
Source: PLoS Negl Trop Dis. 2018 Mar 5;12(3):e0006327. doi: 10.1371/journal.pntd.0006327 (PMC5854421; doi:10.1371/journal.pntd.0006327)
Supplement: S5 Fig — A, Time; B, Singlets; C, Lymphocytes were selected for their size and complexity; D, Selection of viable cells; E, Selection of CD3+ cells; F, Dual labelling for CD4 and CD25 to define CD4+CD25+ cells; G, Selection of CD4+CD25+ cells that do not express CD127; H, Selection, from the CD25+CD4+CD127- population, of lymphocytes co-expressing FOXP3 and CD39; H1, CD4+CD25+CD127-CD39+FOXP3- T cells; H2, CD4+CD25-CD127+CD39+FOXP3+ T cells; H3, CD4+CD25+CD127-CD39-FOXP3+ T cells. Expression of intracellular CTLA-4 (I), OX-40 (J), LAP-TGF-β (L), GITR (M), and LAG-3 (N) was evaluated as shown. (DOCX) [file pntd.0006327.s005.docx]

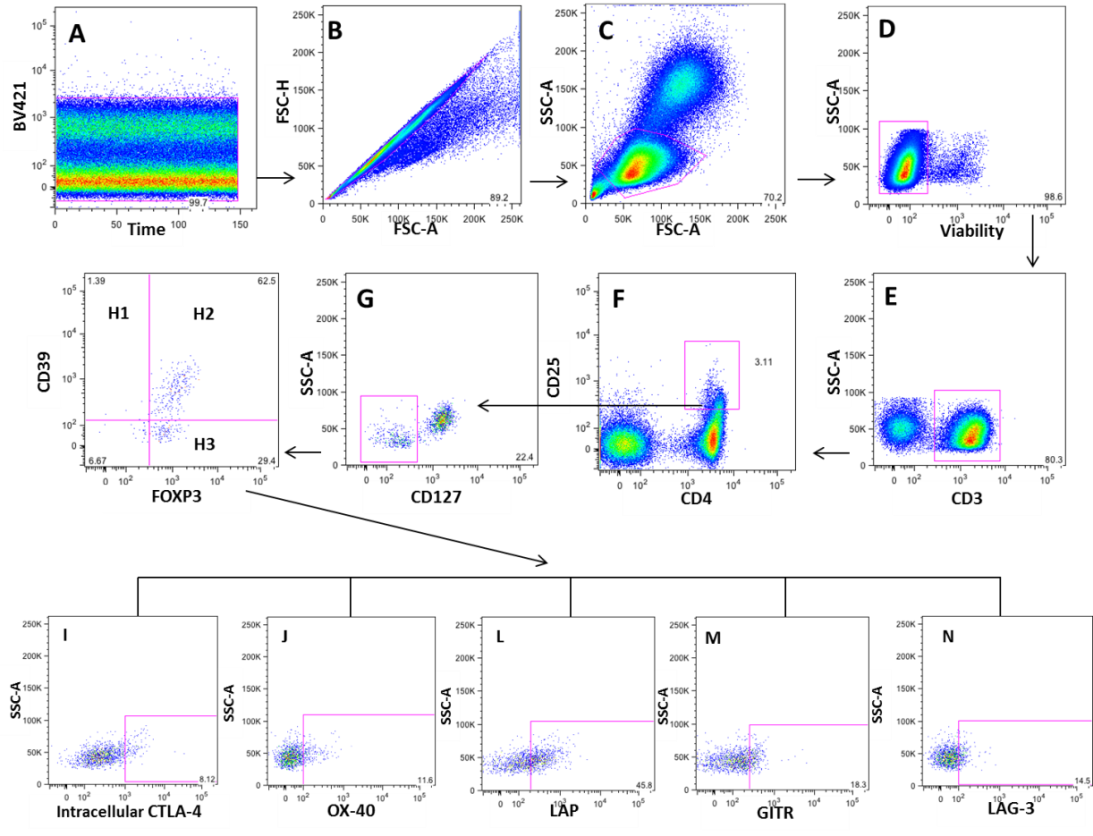


**S5 Fig. Gating strategy to define CD4^+^ T cell subpopulations (co)expressing CD39, FOXP3 and intracellular CTLA-4, OX-40, TGF-β-LAP, GITR, and LAG-3.** A, Time; B, Singlets; C, Lymphocytes were selected for their size and complexity; D, Selection of viable cells; E, Selection of CD3^+^ cells; F, Dual labelling for CD4 and CD25 to define CD4^+^CD25^+^ cells; G, Selection of CD4^+^CD25^+^ cells that do not express CD127; H, Selection, from the CD25^+^CD4^+^CD127^-^ population, of lymphocytes coexpressing FOXP3 and CD39; H1, CD4^+^CD25^+^CD127^-^CD39^+^FOXP3^-^ T cells; H2, CD4^+^CD25^-^CD127^+^CD39^+^FOXP3^+^ T cells; H3, CD4^+^CD25^+^CD127^-^CD39^-^FOXP3^+^ T cells. Expression of intracellular CTLA-4 (I) OX-40 (J), LAP-TGF-β (L), GITR (M) and LAG-3 (N) was evaluated as shown.
