# Supplementary material for: CD39 and immune regulation in a chronic helminth infection: The puzzling case of Mansonella ozzardi
Source: PLoS Negl Trop Dis. 2018 Mar 5;12(3):e0006327. doi: 10.1371/journal.pntd.0006327 (PMC5854421; doi:10.1371/journal.pntd.0006327)
Supplement: S6 Fig — We compared the frequencies of CD39+ and CD39- Treg cells (defined as CD4+CD25hiCD127-FoxP3+ T cells) that expressed a range of regulatory and activation markers. Data are shown for 48 Fil+ and 33 Fil- subjects and were compared using the Wilcoxon signed rank test. Only significant P values after controlling for a false discovery rate (q) set at 0.10 (m = 9) are shown. (DOCX) [file pntd.0006327.s006.docx]

**
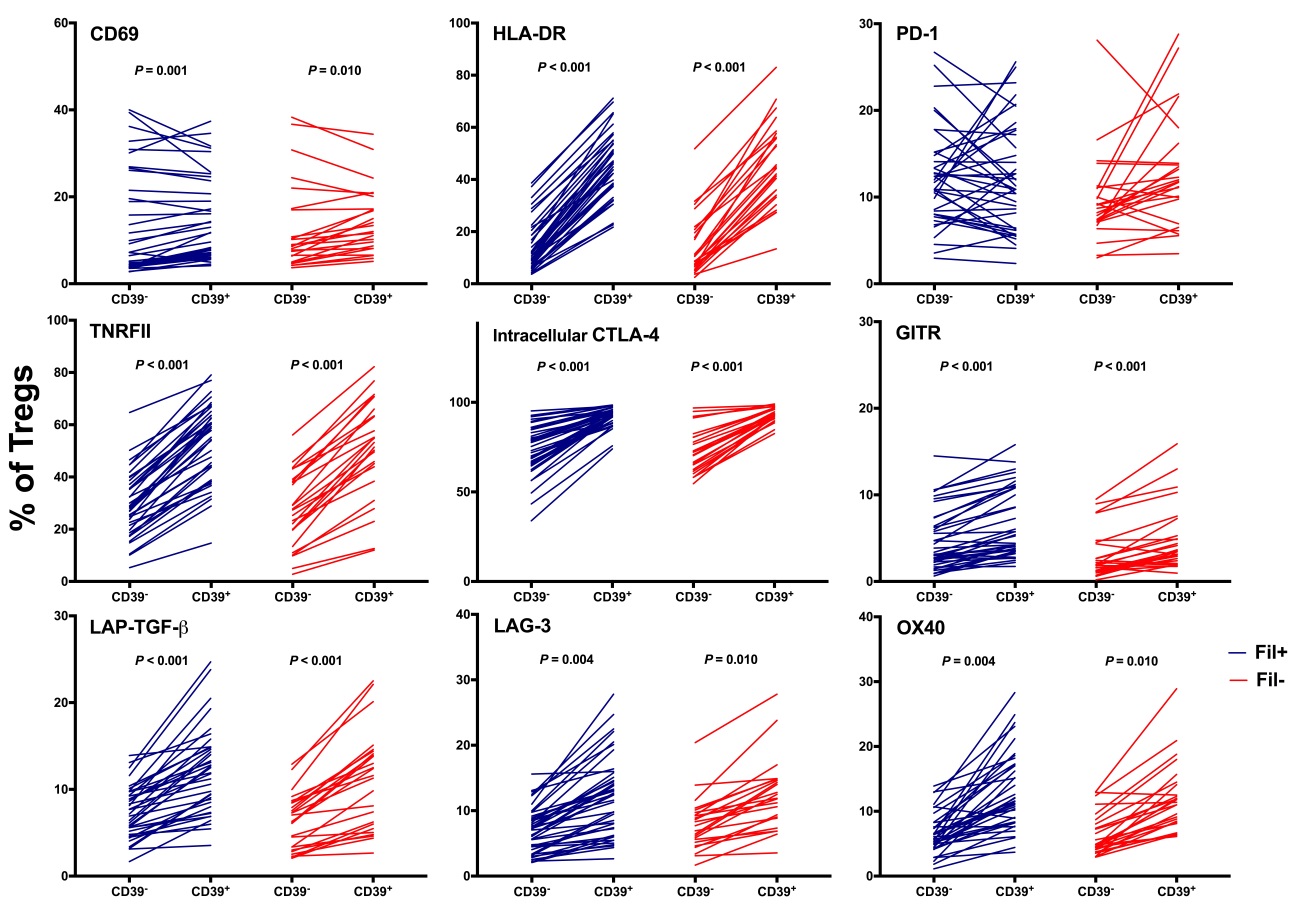
**

**S6 Fig. CD39^+^ Treg cells from microfilaremics and uninfected controls more often express intracellular CTLA-4, LAP-TGB-β, LAG3, TNFRII, GITR, OX40, HLA-DR, and CD69 (but not PD1) than CD39^-^ Treg cells.** We compared the frequencies of CD39^+^ and CD39^-^ Treg cells (defined as CD4^+^CD25^hi^CD127^-^FoxP3^+^ T cells) that expressed a range of regulatory and activation markers. Data are shown for 48 Fil+ and 33 Fil- subjects and were compared using the Wilcoxon signed rank test. Only significant *P* values after controlling for a false discovery rate (*q*) set at 0.10 (*m* = 9) are shown.
