# Supplementary material for: CD39 and immune regulation in a chronic helminth infection: The puzzling case of Mansonella ozzardi
Source: PLoS Negl Trop Dis. 2018 Mar 5;12(3):e0006327. doi: 10.1371/journal.pntd.0006327 (PMC5854421; doi:10.1371/journal.pntd.0006327)
Supplement: S7 Fig — PBMC from microfilaremic (Fil+) and uninfected (Fil-) subjects were stimulated with Staphylococcus aureus enterotoxin B (SEB) in the presence or absence of anti-CD39 antibody, stained for intracellular cytokines, and then incubated with 2mM adenosine. The % of CD4+ T cells producing each cytokine was estimated by flow cytometry. Data are presented for 11 Fil+ and 5 Fil- subjects and were compared using the Wilcoxon signed rank test. Only significant P values after controlling for a false discovery rate (q) set at 0.10 (m = 5 for each group [Fil+ and Fil-] and each pair of experimental conditions [SEB vs. SEB+anti-CD39, SEB vs. SEB+anti-CD39+adenosine; SEB+anti-CD39 vs. SEB+anti-CD39+adenosine]) are shown. (DOCX) [file pntd.0006327.s007.docx]

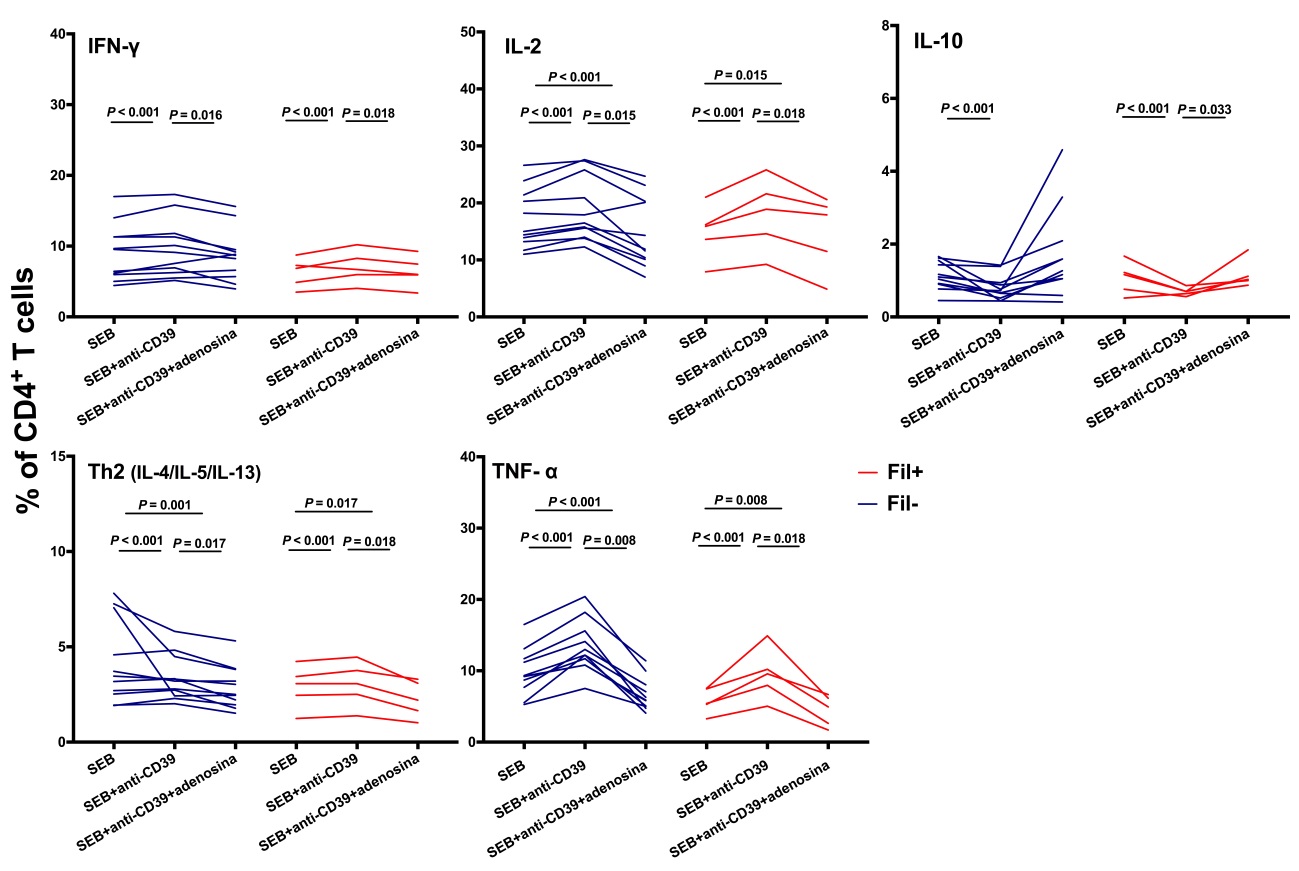


**S7 Fig. Changes in the proportions of CD4^+^ T cells producing IFN-γ, IL-2, and TNF-α, Th2-type cytokines, and IL-10 in the presence of anti-CD39 antibody are reversed in the presence of 2mM adenosine.** PBMC from microfilaremic (Fil+) and uninfected (Fil-) subjects were stimulated with *Staphylococcus aureus* enterotoxin B (SEB) in the presence or absence of anti-CD39 antibody, stained for intracellular cytokines, and then incubated with 2mM adenosine. The % of CD4^+^ T cells producing each cytokine was estimated by flow cytometry. Data are presented for 11 Fil+ and 5 Fil- subjects and were compared using the Wilcoxon signed rank test. Only significant *P* values after controlling for a false discovery rate (*q*) set at 0.10 (*m* = 5 for each group [Fil+ and Fil-] and pair of experimental conditions [SEB vs. SEB+anti-CD39, SEB vs. SEB+anti-CD39+adenosine; SEB+anti-CD39 vs. SEB+anti-CD39+adenosine]) are shown.

.
