# Supplementary material for: CD39 and immune regulation in a chronic helminth infection: The puzzling case of Mansonella ozzardi
Source: PLoS Negl Trop Dis. 2018 Mar 5;12(3):e0006327. doi: 10.1371/journal.pntd.0006327 (PMC5854421; doi:10.1371/journal.pntd.0006327)
Supplement: S1 Table — (PDF) [file pntd.0006327.s008.pdf]

S1 Table. **Panel 1: monoclonal antibodies used to characterize regulatory and activation markers on CD4 $^{+}$ T cells**.

Specificity	Fluorochrome	Volume per test (µL)	Manufacturer
FoxP3	PerCPcy5.5	5	e-Bioscience
CD39	FITC	3	Biolegend
CD4	APCcy7	1	Biolegend
CTLA-4	APC	5	Biolegend
HLA-DR	Alexa 700	3	BD
CD25	BV 605	3	Biolegend
PD-1	BV 650	5	BD Horizon
Viability	Acqua	0.67	Invitrogen
CD127	BV 421	3	Biolegend
CD3	PE-TR	1	Invitrogen
CD69	PEcy7	3	Biolegend
CD8	PEcy5	20	BD
TNFRII	PE	7.5	BD
