# Supplementary material for: CD39 and immune regulation in a chronic helminth infection: The puzzling case of Mansonella ozzardi
Source: PLoS Negl Trop Dis. 2018 Mar 5;12(3):e0006327. doi: 10.1371/journal.pntd.0006327 (PMC5854421; doi:10.1371/journal.pntd.0006327)
Supplement: S2 Table — (PDF) [file pntd.0006327.s009.pdf]

S2 Table. **Panel 2: Monoclonal antibodies used to characterize regulatory and activation markers on $CD4^+$ T cells.**

Specificity	Fluorochrome	Volume per test (μL)	Manufacturer
FoxP3	Percpcy5.5	5	e-Bioscience
CD39	FITC	3	Biolegend
CD4	APCcy7	1	Biolegend
CTLA-4	APC	5	Biolegend
CD25	BV 605	3	Biolegend
OX40	BV 650	5	BD horizon
Viability	Acqua	0.67	Invitrogen
CD127	BV 421	3	Biolegend
CD3	PE-TR	1	Invitrogen
LAG3	PEcy7	3	eBiosciences
GITR	PEcy5	5	Biolegend
LAP-TGF-β	PE	3	Biolegend
