# Supplementary material for: CD39 and immune regulation in a chronic helminth infection: The puzzling case of Mansonella ozzardi
Source: PLoS Negl Trop Dis. 2018 Mar 5;12(3):e0006327. doi: 10.1371/journal.pntd.0006327 (PMC5854421; doi:10.1371/journal.pntd.0006327)
Supplement: S3 Table — (PDF) [file pntd.0006327.s010.pdf]

S3 Table. Panel 3: Monoclonal antibodies used to characterize Ki67-expressing Treg cells.

Specificity	Fluorochrome	Volume per test (µL)	Manufacturer
FOXP3	Percpcy5.5	5	eBioscience
CD39	BV421	0.5	Biolegend
CD4	PE	1	Biolegend
CD3	Pecy7	1	Biolegend
CD127	APCcy7	1	eBioscience
CD25	FITC	0.5	Biolegend
Ki67	APC	2	BD
Viability	Acqua	0.67	Invitrogen
