# Supplementary material for: CD39 and immune regulation in a chronic helminth infection: The puzzling case of Mansonella ozzardi
Source: PLoS Negl Trop Dis. 2018 Mar 5;12(3):e0006327. doi: 10.1371/journal.pntd.0006327 (PMC5854421; doi:10.1371/journal.pntd.0006327)
Supplement: S4 Table — (PDF) [file pntd.0006327.s011.pdf]

S4 Table. Panel 4: Monoclonal antibodies used for intracellular cytokine staining in $CD4^+$ T cells.

Specificity	Fluorochrome	Volume per test (µL)	Manufacturer
CD3	APCcy7	0.5	Biolegend
CD4	PerCPcy5.5	0.5	Biolegend
IL-2	FITC	0.25	Biolegend
IL-4	PE	0.5	EBiociences
IL-5	PE	0.5	BD
IL-10	APC	1	Biolegend
IL-13	PE	1	BD
IFN-Y	BV421	0.5	Biolegend
Viability	Acqua	0.67	Invitrogen
TNF-α	Pecy7	0.25	Biolegend
