# Supplementary material for: CD39 and immune regulation in a chronic helminth infection: The puzzling case of Mansonella ozzardi
Source: PLoS Negl Trop Dis. 2018 Mar 5;12(3):e0006327. doi: 10.1371/journal.pntd.0006327 (PMC5854421; doi:10.1371/journal.pntd.0006327)
Supplement: S5 Table — (PDF) [file pntd.0006327.s012.pdf]

**S5 Table. Frequency of clinical signs and symptoms reported by *M. ozzardi*-infected subjects and uninfected controls.**

| Symptoms | % (number) per group | | *P* value |
|---|---|---|---|
| | fil- | fil+ | |
| No. of subjects | 34 | 50 | |
| Presence of any symptom | 50 (17) | 54 (27) | 0.445 |
| Fever | 5.9 (2) | 2.0 (1) | 0.161 |
| Headache | 20.6 (7) | 14.0 (7) | 0.307 |
| Myalgia | 11.8 (4) | 16.0 (8) | 0.416 |
| Arthralgia | 20.6 (7) | 14.0 (7) | 0.307 |
| Dizziness | 2.9 (1) | 6.0 (3) | 0.465 |
| Weakness | 2.9 (1) | 6.0 (3) | 0.465 |
| Abdominal pain | 2.9 (1) | 16.0 (8) | 0.056 |
| Vomiting | 0.0 (0) | 2.0 (1) | 0.595 |

Proportions were compared with the $\chi^2$ test.
