# Supplementary material for: CD39 and immune regulation in a chronic helminth infection: The puzzling case of Mansonella ozzardi
Source: PLoS Negl Trop Dis. 2018 Mar 5;12(3):e0006327. doi: 10.1371/journal.pntd.0006327 (PMC5854421; doi:10.1371/journal.pntd.0006327)
Supplement: S6 Table — Subjects with BmA antibody levels above the overall median were defined as IgG4H, while those with BmA antibody levels below the overall median were defined as IgG4L. (PDF) [file pntd.0006327.s013.pdf]

S6 Table. Demographic, hematologic, and clinical characteristics of study participants according to levels of BmA-specific IgG_4 antibodies. Subjects with BmA antibody levels above the overall median were defined as IgG4H, while those with BmA antibody levels below the overall median were defined as IgG4L.

Characteristic	Value for group		*P* value
	IgG4L	IgG4H	
No. of subjects	56	26	
Age in years (range)	37 (7-89)	44.(12-98)	0.199
Gender (% male)	46.4	69.2	0.284
Village			
Boa Vista	8	0	0.014
Monte Verde	11	5	
Nova Vida	15	5	
Retiro	11	6	
São Pedro	2	2	
Valparaíso	7	8	
Hemoglobin levels (g/100 mL)	14.1 (11.8-21.2)	14.9 (11.7-17.40)	0.059
Anemia (%)	2.9 (n = 1)	6.0 (n = 3)	0.410
No. of WBCs (10^9/L)	8.1 (3.9-17)	7.9 (3.9-15.4)	0.668
No. of lymphocytes (10^9/L)	2.6 (1.3-5.6)	2.0 (1.2-3.8)	0.009
No. of granulocytes (10^9/L)	4.6 (1.9-13.7)	5.0 (1.9-43)	0.069
No. of T lymphocytes (10^9/L)	1.74 (0.90-3.58)	1.42 (0.66-2.75)	0.009
No. of $CD4^+$ T lymphocytes (10^9/L)	1.05 (0.43-2.13)	0.80 (0.43-1.70)	0.008

Data are presented as median (interquartile range) except if otherwise indicated and were compared with the Mann-Whitney test (continuous variables) or with the χ^2 test (proportions).
