# Supplementary material for: CD39 and immune regulation in a chronic helminth infection: The puzzling case of Mansonella ozzardi
Source: PLoS Negl Trop Dis. 2018 Mar 5;12(3):e0006327. doi: 10.1371/journal.pntd.0006327 (PMC5854421; doi:10.1371/journal.pntd.0006327)
Supplement: S7 Table — (PDF) [file pntd.0006327.s014.pdf]

S7 Table. Levels of plasma cytokines in microfilaremic subjects (Fil+) and uninfected controls (Fil-).

Cytokine	Value for group (pg/mL)		*P* value
	Fil-	Fil+	
No. of subjects	34	49	
IL-1ra	53.30 (21.60-588.87)	46.17 (0-166.41)	0.212
TNF-α	18.34 (0-36.42)	9.73 (0-56.09)	0.012*
IL-2	Not detected	Not detected	NA
IL-7	29.05 (0-68.09)	17.78 (0-84.98)	0.029
IP10	26.00 (6.56-89.23)	22.27 (11.86-130.06)	0.869
IL-2r	206.44 (78.54-469.40)	236.01 (0-510.32)	0.157
MIG	215.20 (17.06-2355.04)	137.28 (26.83-2375.68)	0.435
IL-4	9.25 (0.00-14.23)	0.00 (0.00-88.11)	0.008*
IL-8	28.53 (0.00-43.24)	4.68 (0.00-268.24)	0.005*
FGF	2.70 (0.00-190.69)	6.11 (0.00-11.01)	0.465
IL-1β	0.45 (0.00-21.89)	0.44 (0.00-13.33)	0.720
G-CSF	101.86 (0.00-302.16)	52.36 (0.00-487.96)	0.016*
IL-10	13.69 (0.00-653.05)	17.45 (0.00-67.01)	0.052
IL-13	29.08 (0.00-182.97)	10.80 (0.00-161.32)	0.098
IL-6	7.99 (0.00-48.90)	3.85 (0.00-34.69)	0.021*
IL-12 (p40/p70)	126.98 (47.56-270.87)	118.08 (60.32-359.40)	0.566
CCL5/RANTES	230.63 (0.00-1268.45)	303.80 (0.00-1293.65)	0.501
Eotaxin	37.00 (13.10-93.89)	47.72 (18.21-66.05)	0.006*
IL-17	2.25 (0-4.45)	0.00 (0.00-35.06)	0.064
MIP-1α	28.92 (0.00-108.29)	16.05 (0.00-84.77)	0.016*
GM-CSF	Not detected	Not detected	NA
MIP-1β	54.36 (0.00-128.88)	31.83 (0.00-84.77)	0.034
MCP-1	121.61 (48.38-561.06)	144.24(57.52-223.26)	0.198
IL-15	31.51 (0.00-556.68)	22.23 (0.0-199.02)	0.034
EGF	4.64 (0.00-66.99)	3.34 (0.00-23.52)	0.150
IL-5	7.90 (0.00-48.92)	5.40 (0.00-30.49)	0.176
HGF	61.81 (21.60-158.82)	52.54 (24.56-136.63)	0.110
VEGF	0.93 (0.00-11.85)	0.57 (0.00-13.96)	0.836
IFN-ϒ	8.30 (0.00-46.40)	3.99 (0.00-42.37)	0.117
IFN-α	37.64 (0.00-434.04)	38.44 (0.00-89.40)	0.356

Data are presented as medians (interquartile ranges) and were compared with the Mann-Whitney *U* test. * indicates a significant result after controlling for a false discovery rate (*q*) set at 0.10 (*m* = 28). NA = not applicable.
