# Supplementary material for: CD39 and immune regulation in a chronic helminth infection: The puzzling case of Mansonella ozzardi
Source: PLoS Negl Trop Dis. 2018 Mar 5;12(3):e0006327. doi: 10.1371/journal.pntd.0006327 (PMC5854421; doi:10.1371/journal.pntd.0006327)
Supplement: S8 Table — (PDF) [file pntd.0006327.s015.pdf]

S8 Table. Proportion of $CD4^+$ T cells from microfilaremic subjects (Fil+) and uninfected controls (Fil-) producing specific cytokines upon stimulation *in vitro* with filarial (BmA) and unrelated (SEB) antigen.

Citokine	Value for group (% of $CD4^+$ cells)			
	**BmA**		**SEB**	
	Fil-	Fil+	Fil-	Fil+
No. subjects	28	40	28	40
IFN-Y	0.15 (0.09-0.32)	0.18 (0.09-0.40)	6.83 (4.72-8.86)	7.4 (4.93-10.22)
IL-2	0.35 (0.23-0.57)	0.34 (0.19-0.51)	16.35 (13.37-19.82)	15.95 (11.77-20.25)
IL-10	0.78 (0.27-1.51)	1.08 (0.50-1.94)	0.94 (0.70-1.40)	1.00 (0.73-1.73)
Th2	0.21 (0.11-0.41)	0.17 (0.13-0.22)	3.06 (2.31-3.91)	3.45 (2.52-4.41)
TNF-α	0.21 (0.13-0.30)	0.18 (0.14-0.32)	9.27 (6.02-10.52)	9.26 (7.36-12.30)

Data are presented as medians (interquartile ranges) and were compared with the Mann-Whitney *U* test. No significant difference was found between cells from Fil+ and Fil- subjects stimulated with the same antigen after controlling for a false discovery rate (q) = 0.10, m = 5. Th2 = IL-4, IL-5, and IL-13
