# Supplementary material for: CD39 and immune regulation in a chronic helminth infection: The puzzling case of Mansonella ozzardi
Source: PLoS Negl Trop Dis. 2018 Mar 5;12(3):e0006327. doi: 10.1371/journal.pntd.0006327 (PMC5854421; doi:10.1371/journal.pntd.0006327)
Supplement: S9 Table — (PDF) [file pntd.0006327.s016.pdf]

S9 Table. **Levels of cytokines in PBMC culture supernatants from microfilaremic subjects (Fil+) and uninfected controls (Fil-) after stimulation *in vitro* with filarial (BmA) and unrelated (SEB) antigen.**

	Citokine concentration for group (pg/mL)			
	**BmA**		**SEB**	
	Fil-	Fil+	Fil-	Fil+
No. subjects	15	27	15	27
**IL-6**	876.24 (450.68-2345.21)	1329.37 (657.10-3751.14)	4299.47 (1435.60-6386.87)	6539.97 (2239.49-10376.40)
**IL-13**	311.01 (132.51-469.32)	387.30 (65.15-1677.16)	2110.80 (877.52-2846.52)	2382.2 (843.21-3126.13)
**IL-10**	132.38 (27.00-350.84)	174.37 (67.09-611.78)	556.92 (143.00-759.48)	1176.39 (147.33-1301.28)
**IL-4**	3.26 (1.23-29.69)	2.73 (1.15-30.82)	59.48 (11.00-177.32)	122.23 (35.00-238.90)
**IFN-Y**	213.90 (88.65-181.74)	184.34 (76.65-165.43)	827.37 (234.93-1134.28)	926.26 (364.18-1478.63)

Data are presented as medians (interquartile ranges) and were compared with the Mann-Whitney *U* test. No significant difference was found between levels of cytokines released by cells from Fil+ and Fil- subjects stimulated with the same antigen after controlling for a false discovery rate (q) = 0.10, m = 5.
