# Supplementary material for: CD39 and immune regulation in a chronic helminth infection: The puzzling case of Mansonella ozzardi
Source: PLoS Negl Trop Dis. 2018 Mar 5;12(3):e0006327. doi: 10.1371/journal.pntd.0006327 (PMC5854421; doi:10.1371/journal.pntd.0006327)
Supplement: S10 Table — (PDF) [file pntd.0006327.s017.pdf]

S10 Table. Frequency (%) of T CD4$^+$ lymphocytes expressing regulation and activation markers in study participants divided into IgG4L and IgGH groups according to their levels of BmA-specific IgG$_4$ antibodies.

Marker	Value for group (% of CD4$^+$ T cells)		*P* value
	IgG4L	IgG4H	
No. subjects	52	24	
CD39	5.86 (2.59-7.70)	7.56 (6.09-11.40)	0.005*
CTLA-4	0.59 (0.04-9.84)	0.41 (0.06-7.15)	0.275
Intracellular CTLA-4	21.40(16.23-42.81)	20.65 (12.80-27.10)	0.621
HLADR	4.44 (1.78-12.60)	5.06 (3.10-11.30)	0.229
PD1	9.34 (3.18-12.50)	8.39 (2.67-11.80)	0.356
TNFRII	5.73 (1.45-16.50)	6.54 (2.03-15.60)	0.240
GITR	0.37 (0.11-2.00)	0.62 (0.13-2.11)	0.015
LAG3	0.08 (0.02-0.47)	0.10 (0.02-0.78)	0.954
LAP	4.04 (1.54-13.20)	4.36 (2.35-12.40)	0.463
OX40	8.50 (3.68-16.80)	7.72 (2.78-22.10)	0.653
CD69	4.62 (1.60-18.50)	7.32 (2.15-18.70)	0.362

Data are presented as medians (interquartile ranges) and were compared with the Mann-Whitney *U* test . * indicates a significant result after controlling for a false discovery rate (q) set at 0.10 (m = 11).
