# Supplementary material for: CD39 and immune regulation in a chronic helminth infection: The puzzling case of Mansonella ozzardi
Source: PLoS Negl Trop Dis. 2018 Mar 5;12(3):e0006327. doi: 10.1371/journal.pntd.0006327 (PMC5854421; doi:10.1371/journal.pntd.0006327)
Supplement: S11 Table — (PDF) [file pntd.0006327.s018.pdf]

S11 Table. Frequency (%) of $CD4^{+}CD25^{hi}CD127^{-}FoxP3^{+}$ (Treg) cells expressing regulatory and activation markers in study participants divided into IgG4L and IgGH groups according to their levels of BmA-specific IgG_4 antibodies.

Marker	Value for group (% of Treg cells)		*P* value
	IgG4L	IgG4H	
No. of subjects	52	24	
$CD69^{+}$	3.17 (1.18-6.50)	3.32 (0.89-7.90)	0.350
$CTLA\text{-}4^{+}$	0.40 (0.01-2.30)	0.94 (0.02-3.18)	0.542
$CTLA\text{-}4^{+}$ intracelular	80.20 (68.90-90.30)	77.10 (65.90-87.60)	0.244
$HLADR^{+}$	30.60 (18.20-35.20)	26.65 (12.31-45.30)	0.117
$PD1^{+}$	4.46 (1.53-8.80)	4.22 (1.90-7.30)	0.950
$TNFRII^{+}$	45.40 (28.76-56.90)	42.25 (27.50-61.30)	0.972
$GITR^{+}$	0.65 (0.01-0.79)	0.33 (0.01-0.59)	0.058
$LAG3^{+}$	0.15 (0.00-0.32)	0.14 (0.00-0.26)	0.718
LAP^{+}	7.17 (3.91-14.70)	6.10 (2.35-12.00)	0.470
$OX40^{+}$	3.48 (1.63-6.33)	3.84 (1.86-5.54)	0.784
CD39+	59.90 (48.05-64.40)	68.2 (58.3-70.16)	0.022
$CD39^{+}CD69^{+}$	3.09 (1.44-8.90)	3.42 (0.80-9.50)	0.395
$CD39^{+}CTLA\text{-}4^{+}$	0.40 (0.01-1.31)	1.06 (0.10-1.52)	0.939
$CD39^{+}$intracellular CTLA-4+	84.00 (68.90-88.20)	83.90 (71.35-86.60)	0.766
$CD39^{+}HLADR^{+}$	35.10 (21.30-49.80)	32.45 (23.70-54.30)	0.595
$CD39^{+}PD1^{+}$	3.50 (0.70-8.10)	3.76 (0.94-8.70)	0.603
$CD39^{+}TNFRII^{+}$	46.10 (22.10-53.80)	45.75 (21.62-50.10)	0.814
$CD39^{+}GITR^{+}$	0.49 (0.01-0.98)	0.36 (0.00-0.52)	0.275
$CD39^{+}LAG3^{+}$	0.13 (0.00-0.25)	0.13 (0.00-0.22)	0.757
$CD39^{+}LAP^{+}$	6.82 (1.18-13.10)	6.16 (2.10-12.40)	0.879
$CD39^{+}OX40^{+}$	4.17 (1.71-6.82)	4.55 (2.01-6.76)	0.780
$CD39^{-}CD69^{+}$	4.22 (1.53-11.30)	2.86 (0.45-9.40)	0.347
$CD39^{-}CTLA4^{+}$	0.48 (0.00-2.86)	1.10 (0.12-1.99)	0.461
$CD39^{-}$intracellular $CTLA\text{-}4^{+}$	65.05 (39.40-77.60)	64.20 (37.87-75.60)	0.355

$CD39^{-}HLADR^{+}$	16.30 (9.40-25.20)	11.30 (9.80-14.43)	0.127
$CD39^{-}PD1^{+}$	7.83 (5.32-11.70)	5.70 (3.10-10.40)	0.141
$CD39^{-}TNFRII^{+}$	35.85 (18.80-29.45)	30.30 (13.45-34.90)	0.076
$CD39^{-}GITR^{+}$	0.04 (0.00-0.51)	0.04 (0.00-1.56)	0.083
$CD39^{-}LAG3^{+}$	0.00 (0.00-0.60)	0.00 (0.00-0.98)	0.380
$CD39^{-}LAP^{+}$	5.87 (2.24-13.50)	5.26 (1.76-10.50)	0.708
$CD39^{-}OX40^{+}$	2.54 (0.78-8.20)	2.93 (1.21-9.10)	0.582

Data are presented as medians (interquartile ranges) and were compared with the Mann-Whitney *U* test. No difference remained statistically significant after controlling for a false discovery rate (*q*) = 0.10, *m* = 31.
